# A novel protein encoded by circRsrc1 regulates mitochondrial ribosome assembly and translation during spermatogenesis

**DOI:** 10.1186/s12915-023-01597-z

**Published:** 2023-04-24

**Authors:** Shu Zhang, Chang Wang, Yue Wang, Hao Zhang, Chen Xu, Yiwei Cheng, Yan Yuan, Jiahao Sha, Xuejiang Guo, Yiqiang Cui

**Affiliations:** 1 State Key Laboratory of Reproductive Medicine and Offspring Health, Nanjing Medical University, Nanjing, 211166 China; 2 State Key Laboratory of Reproductive Medicine and Offspring Health, Women’s Hospital of Nanjing Medical University, Nanjing Maternity and Child Health Care Hospital, Nanjing Medical University, Nanjing, 210029 China

**Keywords:** circRsrc1, Translation, Mitochondrial ribosome, C1qbp, Spermatogenesis

## Abstract

**Background:**

Circular RNAs (circRNAs) are a large class of mammalian RNAs. Several protein products translated by circRNAs have been reported to be involved in the development of various tissues and systems; however, their physiological functions in male reproduction have yet not been explored.

**Results:**

Here, we report an endogenous circRNA (circRsrc1) that encodes a novel 161-amino-acid protein which we named Rsrc1-161aa through circRNA sequencing coupled with mass spectrometry analysis on mouse testicular tissues. Deletion of Rsrc1-161aa in mice impaired male fertility with a significant decrease in sperm count and motility due to dysfunctions of mitochondrial energy metabolism. A series of in vitro rescue experiments revealed that circRsrc1 regulates mitochondrial functions via its encoded protein Rsrc1-161aa. Mechanistically, Rsrc1-161aa directly interacts with mitochondrial protein C1qbp and enhances its binding activity to mitochondrial mRNAs, thereby regulating the assembly of mitochondrial ribosomes and affecting the translation of oxidative phosphorylation (OXPHOS) proteins and mitochondrial energy metabolism.

**Conclusions:**

Our studies reveal that Rsrc1-161aa protein encoded by circRsrc1 regulates mitochondrial ribosome assembly and translation during spermatogenesis, thereby affecting male fertility.

**Supplementary Information:**

The online version contains supplementary material available at 10.1186/s12915-023-01597-z.

## Background

Circular RNAs (circRNAs) are single-stranded RNAs with a covalent closed loop structure [1, 2]. Studies have shown that circRNAs can function as microRNA sponges [3], regulate gene transcription and splicing [4, 5], and/or act as scaffolds to interact with proteins [6–9]. Although circRNAs were previously thought to be non-coding RNAs, growing evidence now suggests that some circRNAs can also be translated. For example, *circZNF609*, *circMbl*, and *circFGFR1* have internal ribosome entry sites (IRES) for translation [10–12]. circRNA-encoded microproteins have been identified in the human heart [13]. Proteins translated from *circ-E-Cad*, *circMAPK1*, and *circ-SMO* participate in tumorigenesis [14–16]. However, the physiological functions of proteins encoded by circRNAs in male reproduction remain to be explored.

Studies suggest that circRNAs are abundant in mammalian testis [17, 18] and were found to have differential expression in non-obstructive azoospermia patients, indicating their role in spermatogenesis [19]. Sex-determining region Y (Sry), a testis-specific circRNA, acts as a sponge for miR-138 [3]. About half of the circRNAs contain open reading frames with m6A-modified start codons in mouse male germ cells, indicating their coding potential [20]. Nevertheless, the functions and mechanisms of proteins encoded by circRNAs in spermatogenesis demand further study.

Mitochondria, the energy-producing organelles in cells, can be regulated by circRNAs. mc-COX2 can dampen mitochondrial function and promote cell proliferation [21]. circRNA SCAR was shown to alleviate nonalcoholic steatohepatitis by regulating the permeability of mitochondrial transition pores [22]. In addition to mitochondrial genome-derived circRNAs, nuclear genome-derived circRNAs can also regulate mitochondrial function. For example, circPUM1 participates in the assembly of mitochondrial complex III and the regulation of oxidative phosphorylation [23]. MFACR participates in mitochondrial fission and apoptosis to mediate the death of cardiomyocyte [24]. However, whether the proteins encoded by circRNAs can regulate mitochondrial functions remains unknown.

In this study, we performed circRNA sequencing and proteomic analysis of mouse testes and identified the circRNA circRsrc1, which encodes a 161 amino acids protein named Rsrc1-161aa. The results of this study suggest that Rsrc1-161aa regulates the assembly and translation of mitochondrial ribosomes. Moreover, the loss of function studies showed that Rsrc1-161aa is related to male fertility and lack of Rsrc1-161aa decreases sperm count and motility in mice.

## Results

### Identification of translatable circRNAs in testis

**Fig. 1:**
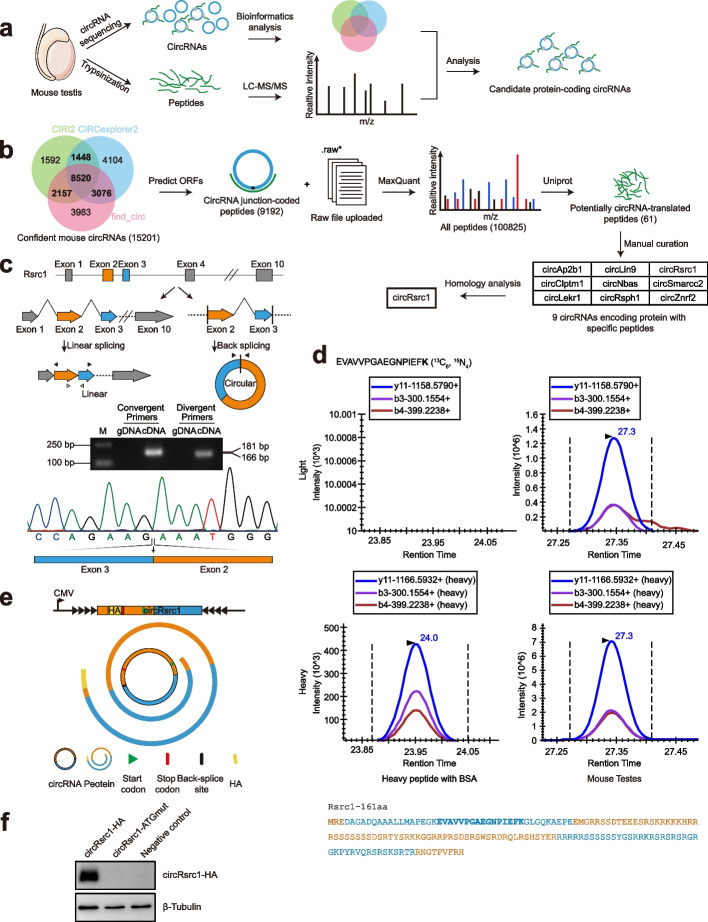
High-throughput sequencing and mass spectrometry of translatable circRNAs in mouse testes. **a** Flow chart of bioinformatic analysis and mass spectrometry detection of translatable circRNAs in mouse testes. **b** Flow chart of the detection of translatable circRNAs in mouse testes. The ORFs of mouse circRNAs were predicted by two or three different algorithms and cross verified by peptide identification using mass spectrometry. The existing Uniprot peptides were removed and manually corrected before human-mouse homology analysis. **c** Illustration of the annotated genomic region of Rsrc1, the putative linear and head-to-tail splicing form, and the validation strategy for the circRsrc1 are shown. Divergent primers (solid arrows) detected the circular form in cDNA but not in gDNA of mouse testes. Convergent primers (hollow arrows) spanning between exons 2 and 3 of Rsrc1 specifically detected the linear splicing form. Sanger sequencing following PCR was performed using the indicated divergent primers. **d** LC–MS/MS analysis was performed to identify specific peptide sequences translated by circRsrc1 in mouse testes. The predicted 161aa peptide sequence is listed; the one in bold blue is the specific peptide identified by mass spectrometry. **e** Diagram of circRsrc1-HA vector; circRsrc1 with an HA tag before stop codon was cloned into a plasmid carrying reverse complementary sequences. **f** Western blotting of cells transfected with circRsrc1-HA or circRsrc1-ATGmut plasmids mutated at the predicted start codon. β-tubulin was used as the internal control

To identify translatable circRNAs in testis, we analyzed circRNAs from the RNA-seq data of circRNAs from mouse testes, and then performed proteomic analysis to identify potential proteins and peptides that could be encoded by circRNAs (Fig. 1a). CIRI2 [25], CIRCexplorer2 [26], and find_circ [1] analyses of circRNAs revealed a total of 15,201 candidate circRNAs that were identified by at least two of the three methods. The open reading frames (ORFs) prediction resulted in 9192 peptides encoded by junction sequences of circRNAs. To further confirm the polypeptides encoded by cirRNAs, we performed proteomic analysis of mouse testicular proteins and peptides by mass spectrometry (MS) and processed mass spectra data by MaxQuant (Ver.1.6.5.0) using predicted ORFs encoded by circRNAs and Uniprot mouse protein sequence as database. After manual curation, we found 9 circRNAs that can encode proteins containing peptides unique to circRNAs (Fig. 1b, Additional file 1: Fig. S1). Reverse transcription-polymerase chain reaction (RT-PCR) and Sanger sequencing confirmed the back-splicing sites of these 9 circRNAs in mouse testes. Among these 9 circRNAs, circRsrc1 of 322 bp in length (circRsrc1^322bp^) is formed by the back-splicing junction between exons 2 and 3 (Fig. 1c, Additional file 1: Fig. S2). Homology analysis showed that only the circRsrc1 is conserved between human and mouse (Fig. 1b).

To confirm the expression of the protein encoded by circRsrc1, we performed targeted MS quantification using heavy isotope-labeled peptide as internal control. The results showed that circRsrc1^322bp^ encodes a 161-amino-acid protein in mouse testes, which we named Rsrc1-161aa (Fig. 1d). Notably, the number of amino acids of Rsrc1-161aa is 161, which is longer than the number of amino acids encoded by 322 bp, because 322 is not the exact multiple of three, and the protein is translated by a rolling circle amplification mechanism. In addition, we evaluated the protein coding potential using a *circRsrc1-HA* vector with flanking sequences of laccase2 [27] to induce circularization (Fig. 1e). Transfection of *circRsrc1-HA* vector in spermatogonia cell line (GC-1 spg) cells resulted in circularization of *circRsrc1-HA* and the expression of about 18 kDa fusion protein circRsrc1-HA. To identify the translation start codon, we mutated the first ATG of putative ORF. Western blotting analysis using anti-HA antibody showed that the ATG mutation prevented the synthesis of circRsrc1-HA protein (Fig. 1f).

### *Rsrc1*^*−/−*^*/circRsrc1*^+*/*+^ mice exhibited normal spermatogenesis

**Fig. 2:**
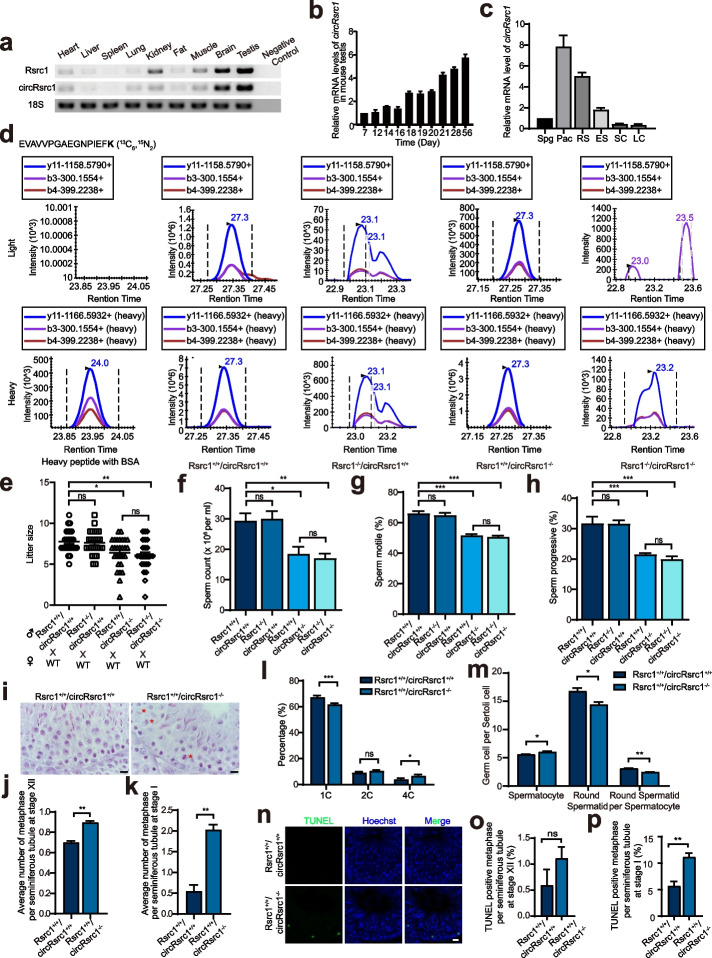
Deletion of Rsrc1-161aa leads to abnormalities in spermatogenesis. **a** Expression levels of Rsrc1 (181 bp) and circRsrc1 (166 bp) were measured by RT-PCR in different mouse tissues. The expression levels are normalized to 18S. **b** Relative expression levels of *circRsrc1* in mouse testes at different ages (in weeks) (*n* = 3). **c** Relative expression levels of *circRsrc1* in different germ cells and somatic cells of mouse testes (*n* = 3). **d** Mass spectrometry detection of *circRsrc1* translated peptides from the wild-type and different knockout mice. **e** The litter size of different knockout and wild-type mice are shown. **f–h** The sperm count, sperm motile, and sperm progressive of different knockout and wild-type mice (*n* = 6). **i** H&E staining of testis sections from adult wild-type and *Rsrc1*^+*/*+^*/circRsrc1*^*−/−*^ mouse. The red asterisk represents the blocked metaphase. Bar, 10 μm. **j,k** Quantification of the average number of metaphases per seminiferous tubule at stage XII and stage I of wild-type and *Rsrc1*^+*/*+^*/circRsrc1*^*−/−*^ mice (*n* = 3). **l** Flow cytometry analysis of testes in wild-type and *Rsrc1*^+*/*+^*/circRsrc1*^*−/−*^ mice (*n* = 13). **m** The counts of spermatocytes and round sperm and the ratio of round sperm to spermatocytes in the testes of wild-type and *Rsrc1*^+*/*+^*/circRsrc1*^*−/−*^ mice (*n* = 3). **n** TUNEL staining of wild-type and *Rsrc1*^+*/*+^*/circRsrc1*^*−/−*^ mice at stage I. Bar, 20 μm. **o,p** Quantification of the proportion of TUNEL positive metaphase per seminiferous tubule in wild-type and *Rsrc1*^+*/*+^*/circRsrc1*.^*−/−*^ mice at stage XII and stage I. All data are means ± SEM. When performing statistical tests between two groups of data, tested using a two-tailed unpaired *t*-test. When performing statistical tests between three or more groups of data, tested using one-way ANOVA with Tukey’s post hoc analysis. *P < 0.05; **P < 0.01; ***P < 0.001

The *Rsrc1* gene is conserved between human and mouse, and it is expressed in various tissues with abundant expression in the testis and brain. Similarly, *circRsrc1* is also highly expressed in the testis and brain, with the highest expression in the testis (Fig. 2a). To further analyze the expression patterns of *circRsrc1* and its parental gene in spermatogenesis, we used reverse transcription quantitative polymerase chain reaction (RT-qPCR) to quantify the levels of transcripts in mouse testes at different developmental stages (Fig. 2b and Additional file 1: Fig. S3a). Consistent with the parental gene, expression of *circRsrc1* began to rise from day 18 after birth and reached the maximum levels from day 21 to adulthood in mouse testis. In addition, we separated spermatogenic cells at different stages by STA-PUT (Sedimentation at Unit Gravity) to quantify the expression levels of *circRsrc1* and *Rsrc1* (Fig. 2c and Additional file 1: Fig. S3b). The results showed that *circRsrc1* is expressed in all stages of spermatogenic cells, including spermatogonia (Spg), spermatocyte (Pac), round spermatid (RS), and elongated sperm (ES), with the highest expression level in spermatocyte. Notably, *circRsrc1* was hardly expressed in the Sertoli cell (SC) or Leydig cell (LC). At the same time, we analyzed the subcellular localization of circRsrc1 in mouse testes by RT-qPCR, and the results showed that circRsrc1 is located in the cytoplasm (Additional file 1: Fig. S3c).

Since there is a partial overlap in protein sequence of protein translated from *circRsrc1* and *Rsrc1*, we first studied the function of *Rsrc1* by constructing the *Rsrc1*^*−/−*^*/circRsrc1*^+*/*+^ mice using CRISPR/Cas9 technology (Additional file 1: Fig. S3d). The genotype of the *Rsrc1*^*−/−*^*/circRsrc1*^+*/*+^ mice was confirmed by polymerase chain reaction (PCR), and RT-PCR analysis showed that *Rsrc1* deletion did not affect the expression of circ*Rsrc1* (Additional file 1: Fig. S3e-f). Meanwhile, Western blotting and targeted MS quantification showed the absence of Rsrc1 protein while circRsrc1 was expressed in *Rsrc1*^*−/−*^*/circRsrc1*^+*/*+^ testis (Fig. 2d and Additional file 1: Fig. S3g).

We found no significant difference in fertility, testicular/ body weight ratio, sperm count, motility, and progressive motility between *Rsrc1*^*−/−*^*/circRsrc1*^+*/*+^ and control mice (Fig. 2e–h and Additional file 1: Fig. S4a). Also, hematoxylin and eosin (H&E) staining of the testis and epididymis confirmed the normal spermatogenesis in the testis and sperm in the *Rsrc1*^*−/−*^*/circRsrc1*^+*/*+^ mice (Additional file 1: Fig. S4d).

### *Rsrc1*^+*/*+^*/circRsrc1*^*−/−*^ mice exhibited abnormal spermatogenesis and oligoasthenozoopermia

To study the in vivo function of circRsrc1, we deleted circRsrc1 without affecting Rsrc1. The flanking complementary sequences in intron play important roles in the formation of circRNA [28]. We used CRISPR/Cas9 system to delete the 85,871-bp intron sequence involved in the formation of *circRsrc1* and generated the *circRsrc1* knockout mice (Additional file 1: Fig. S3h). Genotype analysis by PCR and then Sanger sequencing showed successful deletion of *circRsrc1*^*322bp*^ (Additional file 1: Fig. S3i). RT-PCR analysis confirmed the deletion of *circRsrc1* without affecting the expression of *Rsrc1*. However, RT-PCR showed an additional band of 276 bp (apart from the 128 bp band of *circRsrc1*^*322bp*^) corresponding to *circRsrc1*^*496bp*^ including the exons 2, 3, and 4; *circRsrc1*^*496bp*^ is another isoform of circRsrc1 according to Sanger sequencing (Additional file 1: Fig. S3j). Notably, *circRsrc1*^*496bp*^ was almost invisible but was significantly upregulated after the deletion of *circRsrc1*^*322bp*^. Since Rsrc1-161aa encoded by *circRsrc1*^*322bp*^ is also a part of Rsrc1-321aa (encoded by circRsrc1^496bp^), PRM-based MS targeted quantification still detected the expression of Rsrc1-161aa in the testis of *Rsrc1*^+*/*+^*/circRsrc1*^*−/−*^ mice (Fig. 2d). Concisely, Rsrc1-161aa knockout resulted in a compensatory activation of Rsrc1-321aa encoded by circRsrc1^496bp^.

Because of the compensatory activation of Rsrc1-321aa in *Rsrc1*^+*/*+^*/circRsrc1*^*−/−*^ mice, we further generated the *Rsrc1*^*−/−*^*/circRsrc1*^*−/−*^ mice having the deletion of 19 bp sequence surrounding the splice junction of exon 3 using CRISPR/Cas9 (Additional file 1: Fig. S3k). RT-PCR and RT-qPCR analysis confirmed the successful knockout of both *circRsrc1*^*496bp*^ and *circRsrc1*^*322bp*^ (Additional file 1: Fig. S3l-m). Targeted MS quantification using heavy peptide of the common sequence of Rsrc1-161aa and Rsrc1-321aa showed their absence in the testes of *Rsrc1*^*−/−*^*/circRsrc1*^*−/−*^ mice (Fig. 2d). Also, Western blotting indicated the truncation of Rsrc1 encoded by the parental gene (Additional file 1: Fig. S3n).

Subsequently, we performed the phenotypic analysis and found that both *Rsrc1*^+*/*+^*/circRsrc1*^*−/−*^ and *Rsrc1*^*−/−*^*/circRsrc1*^*−/−*^ mice had successful sperm generation in the testis and epididymis, and normal sperm morphology and testicular/body weight ratio (Additional file 1: Fig. S4a, S4d). However, both of the knockout mice showed male subfertility with a similar level of decrease in fertility (Fig. 2e); sperm counts, motility, and progressive motility were all significantly reduced to a similar lower level (Fig. 2f–h). Concisely, Rsrc1 is dispensable for spermatogenesis but mice lacking *circRsrc1*^*322bp*^ alone or both *circRsrc1*^*496bp*^ and *circRsrc1*^*322bp*^ had similar spermatogenesis defects. *circRsrc1*^*322bp*^ but not *circRsrc1*^*496bp*^ is important for spermatogenesis. Therefore, we used *Rsrc1*^+*/*+^*/circRsrc1*^*−/−*^ mice with Rsrc1-161aa knockout for subsequent studies.

To understand the reason for reduced sperm count in the *Rsrc1*^+*/*+^*/circRsrc1*^*−/−*^ testis, we performed H&E staining. The results showed that *Rsrc1*^+*/*+^*/circRsrc1*^*−/−*^ mice had meiotic arrest with residual meiotic metaphase in Stage I and significantly increased meiotic metaphase spermatocytes in both Stage XII and Stage I (Fig. 2i–k). We further evaluated the ratio of different testicular cells using flow cytometry and found that compared with wild-type mice, the ratio of haploid spermatids decreased in knockout mice (Fig. 2l). Also, the number of spermatocytes increased and round spermatids decreased (Fig. 2m). These results indicated that meiosis was partially blocked in *Rsrc1*^+*/*+^*/circRsrc1*^*−/−*^ mice. To analyze if the germ cell decrease was due to apoptosis, we performed TUNEL assays. We found no significant change in the number of apoptotic cells in the testes of *Rsrc1*^+*/*+^*/circRsrc1*^*−/−*^ mice (Additional file 1: Fig. S4b-c). Also, TUNEL staining showed no significant change of apoptosis in Stage XII; however, Stage I exhibited an increase in apoptotic metaphase germ cells in the testes of *Rsrc1*^+*/*+^*/circRsrc1*^*−/−*^ mice (Fig. 2n–p). These results indicated that apoptosis increased in the delayed meiotic metaphase of Stage I, which potentially reduced the sperm count in *Rsrc1*^+*/*+^*/circRsrc1*^*−/−*^ mice.

### Rsrc1-161aa regulates cell proliferation

To directly investigate the function of Rsrc1-161aa, we evaluated the expression of endogenous *circRsrc1* in GC-1 spg cells. We found increased expression of *circRsrc1* in GC-1 spg cells than in the spermatocyte cell line (GC-2spd(ts)) and therefore used GC-1 spg cells for subsequent cell experiments (Additional file 1: Fig. S5a). Sanger sequencing and PRM quantification revealed that circRsrc1 was endogenously expressed both at the RNA and protein levels in the wild-type *Rsrc1*^+*/*+^*/circRsrc1*^+*/*+^ GC-1 spg cells (Additional file 1: Fig. S5b-c). We then used CRISPR/Cas9 system to remove an intron similar to the generation of *Rsrc1*^+*/*+^*/circRsrc1*^*−/−*^ mice and generated *Rsrc1*^+*/*+^*/circRsrc1*^*−/−*^ GC-1 spg cells (Additional file 1: Fig. S5d-e). The subcellular localization of endogenous *circRsrc1* RNA was analyzed using fluorescence in situ hybridization (FISH) with a junction-specific probe. We found that *circRsrc1* RNA was mainly localized in the cytoplasm of wild-type GC-1 spg cells, while knockout cells showed no signal (Additional file 1: Fig. S5f). Furthermore, PRM quantification and RT-PCR analysis at the RNA and protein levels revealed the absence of *circRsrc1*^*322bp*^ in the knockout GC-1 spg cells but compensatory activation of *circRsrc1*^*496bp*^, which is similar to that in *Rsrc1*^+*/*+^*/circRsrc1*^*−/−*^ mice (Additional file 1: Fig. S5g). The protein encoded by circRsrc1 was still detected in knockout GC-1 spg cells (Additional file 1: Fig. S5c). Rsrc1-321aa encoded by *circRsrc1*^*496bp*^ failed to compensate for the function of Rsrc1-161aa in knockout mice. It is likely that similar to *Rsrc1*^+*/*+^*/circRsrc1*^*−/−*^ mice, *Rsrc1*^+*/*+^*/circRsrc1*^*−/−*^ cells also produced *circRsrc1*^*496bp*^ which encoded Rsrc1-321aa.

**Fig. 3:**
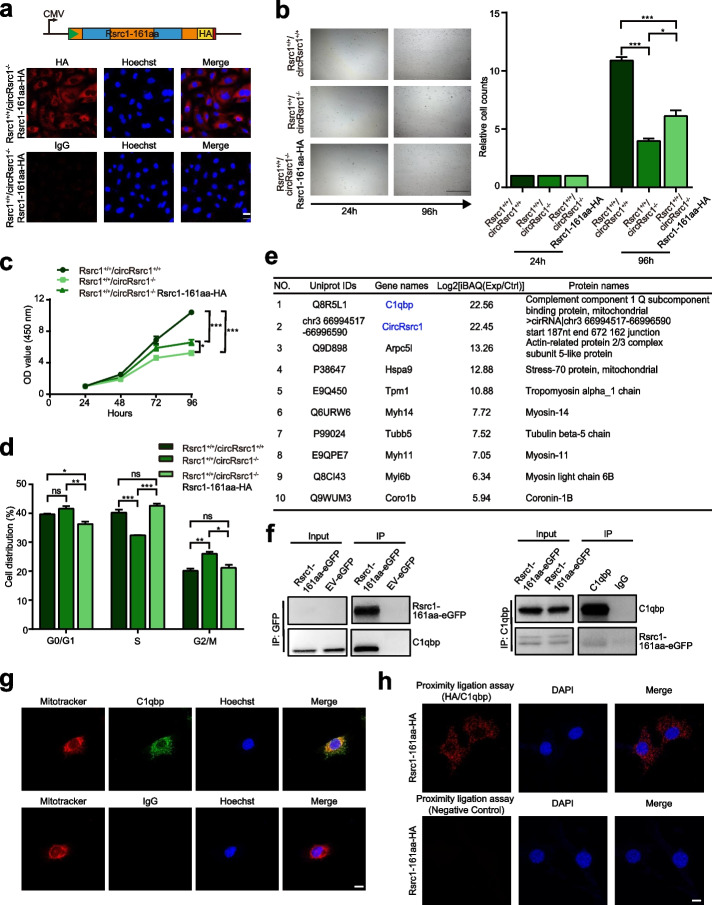
Rsrc1-161aa regulates cell proliferation and cell cycle progression in GC-1 spg cells and interacts with C1qbp. **a** Upper figure: schematic diagram of linearized Rsrc1-161aa. Green triangle, start codon; orange and blue squares, Rsrc1-161aa sequence; yellow square, HA epitope; red square, stop codon. Lower figure: fluorescence images of *Rsrc1*^+*/*+^*/circRsrc1*^*−/−*^ GC-1 spg cells stably transfected with plasmids expressing Rsrc1-161aa. Bar, 20 μm. **b** The cell growth potential was determined based on cell number in wild-type, *Rsrc1*^+*/*+^*/circRsrc1*^*−/*^.^*−*^ and rescue cells. Bar, 400 μm (*n* = 27). **c** The effect of Rsrc1-161aa on cell proliferation by CCK-8 assay (n = 5). **d** The regulatory effect of Rsrc1-161aa on cell cycle progression (*n* = 3). **e** Rsrc1-161aa related proteins were identified by mass spectrometry after immunoprecipitation using anti-GFP antibodies and silver staining. **f** Western blotting of co-immunoprecipitated proteins. **g** Representative confocal microscopy images of GC-1 spg cells after staining with C1qbp (green) and Mitotracker (red). Bar, 10 μm. **h** Representative immunofluorescence images of GC-1 spg cells transiently transfected with Rsrc1-161aa-HA with PLA signal for detection of HA–C1qbp interaction (red). Nuclei were stained with DAPI (blue). Bar, 10 μm. All data are means ± SEM, tested using one-way ANOVA with Tukey’s post hoc analysis *P < 0.05; **P < 0.01; ***P < 0.001

Subsequently, we overexpressed Rsrc1-161aa-HA in *Rsrc1*^+*/*+^*/circRsrc1*^*−/−*^ GC-1 spg cells to better understand the function of Rsrc1-161aa (Fig. 3a, upper figure). As expected, Rsrc1-161aa-HA overexpression was evident by fluorescent signal (Fig. 3a, lower figure). Since *Rsrc1*^+*/*+^*/circRsrc1*^*−/−*^ mice showed a decrease in sperm count, we evaluated the role of circRsrc1 in cell proliferation using *Rsrc1*^+*/*+^*/circRsrc1*^*−/−*^ GC-1 spg cells. As shown in Fig. 3b, compared with the *Rsrc1*^+*/*+^*/circRsrc1*^+*/*+^ GC-1 spg cells, the proliferation of *Rsrc1*^+*/*+^*/circRsrc1*^*−/−*^ GC-1 spg cells was significantly lower. Also, the CCK-8 assay confirmed decreased proliferation of *Rsrc1*^+*/*+^*/circRsrc1*^*−/−*^ GC-1 spg cells (Fig. 3c). Rsrc1-161aa overexpression partially rescued the decreased cell proliferation phenotype (Fig. 3b,c). Meanwhile, TUNEL staining showed no change in apoptosis in *Rsrc1*^+*/*+^*/circRsrc1*^*−/−*^ GC-1 spg cells with or without Rsrc1-161aa overexpression (Additional file 1: Fig. S5h).

To evaluate the role of Rsrc1-161aa in cell cycle progression, we performed cell cycle analysis by measuring DNA content by flow cytometry. The *Rsrc1*^+*/*+^*/circRsrc1*^*−/−*^ cells exhibited G2/M phase arrest with an increase in cells containing 4C DNA, which could be rescued by overexpression of Rsrc1-161aa (Fig. 3d). This indicated that Rsrc1-161aa regulates cell proliferation and G2/M progression, which is consistent with the observed increased of 4C spermatocyte cells in *Rsrc1*^+*/*+^*/circRsrc1*^*−/−*^ mice. Concisely, Rsrc1-161aa regulates the cell cycle progression of spermatogenic cells, and accordingly, *Rsrc1*^+*/*+^*/circRsrc1*^*−/−*^ mice suffered from reduced sperm count.

### Rsrc1-161aa is essential for oxidative phosphorylation (OXPHOS) in mitochondria

To explore the potential molecular mechanism of Rsrc1-161aa in cell proliferation, we identified its interacting proteins by co-immunoprecipitation followed by MS analysis in GC-1 spg cells overexpressing Rsrc1-161aa-eGFP with eGFP as a negative control. Among the identified proteins, we verified C1qbp as the genuine binding partner of Rsrc1-161aa using reciprocal co-immunoprecipitation followed by Western blotting (Fig. 3e,f). Consistent with previous reports [29], we also found that C1qbp was localized to mitochondria (Fig. 3g). To determine a direct interaction between Rsrc1-161aa and C1qbp, we transiently transfected GC-1 spg cells with Rsrc1-161aa-HA plasmid and performed in situ proximity ligation assay (PLA). The results successfully showed the PLA signal in GC-1 spg cells (Fig. 3h), indicating colocalization and direct interaction of Rsrc1-161aa with C1qbp in mitochondria.

**Fig. 4:**
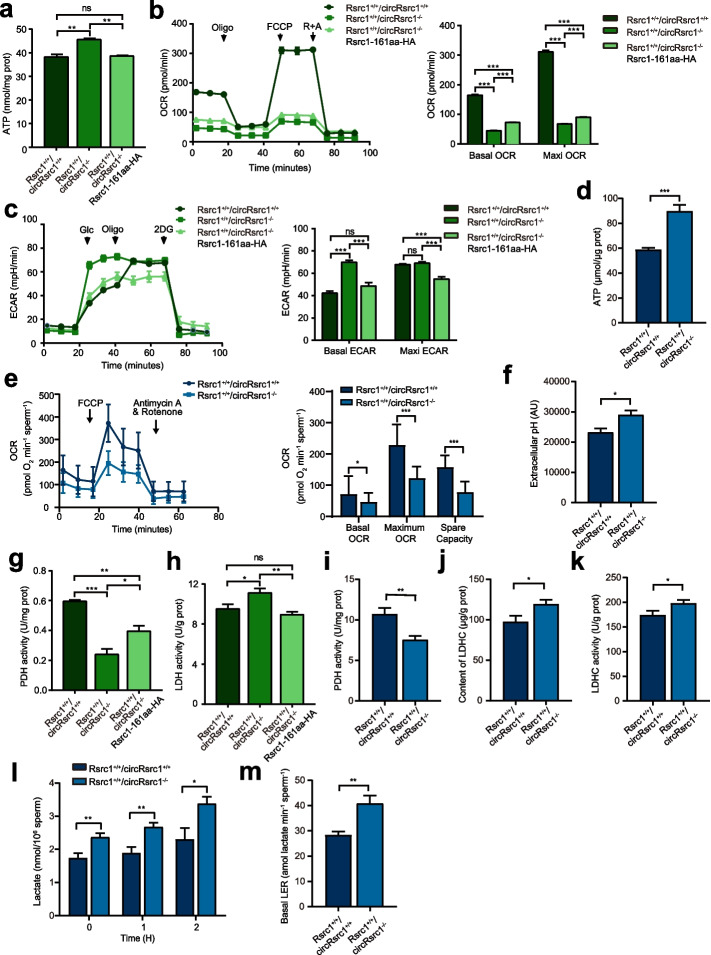
Rsrc1-161aa modulates mitochondrial function. **a** Total ATP levels in wild-type, *Rsrc1*^+*/*+^*/circRsrc1*^*−/−*^ and rescue cells (*n* = 3). **b** Basal and maximal OCRs in wild-type, *Rsrc1*^+*/*+^*/circRsrc1*^*−/−*^ and rescue cells estimated by the XF96 extracellular flux analyzer (*n* = 3). **c** Basal and maximal ECARs in wild-type, *Rsrc1*^+*/*+^*/circRsrc1*^*−/−*^ and rescue cells estimated by the XF96 extracellular flux analyzer (*n* = 3). **d** Total ATP levels in wild-type and *Rsrc1*^+*/*+^*/circRsrc1*^*−/−*^ mice sperm (*n* = 3). **e** Basal and maxim OCRs in wild-type and *Rsrc1*^+*/*+^*/circRsrc1*^*−/−*^ mice sperm (*n* = 3). **f** Extracellular pH value in wild-type and *Rsrc1*^+*/*+^*/circRsrc1*^*−/−*^ mice sperm (*n* = 3). **g** PDH activity in wild-type, *Rsrc1*^+*/*+^*/circRsrc1*^*−/−*^ and rescue cells (*n* = 3). **h** LDH activity in wild-type, *Rsrc1*^+*/*+^*/circRsrc1*^*−/−*^ and rescue cells (*n* = 3). **i** PDH activity in wild-type and *Rsrc1*^+*/*+^*/circRsrc1*^*−/−*^ mice sperm (*n* = 3). **j**,**k** LDHC content and activity in wild-type and *Rsrc1*^+*/*+^*/circRsrc1*^*−/−*^ mice sperm (*n* = 3). **l** Lactate levels were measured in sperm extracts from wild-type and *Rsrc1*^+*/*+^*/circRsrc1*^*−/−*^ mice after 0, 1, and 2 h of incubation in m-TH (*n* = 5). **m** Sperm basal lactate production rate in wild-type and *Rsrc1*^+*/*+^*/circRsrc1*^*−/*^.^*−*^ mice (*n* = 5). All data are means ± SEM. When performing statistical tests between two groups of data, tested using a two-tailed unpaired *t*-test. When performing statistical tests between three or more groups of data, tested using one-way ANOVA with Tukey’s post hoc analysis. *P < 0.05; **P < 0.01; ***P < 0.001

Since C1qbp is known to be associated with OXPHOS in mitochondria [30], we analyzed the similar function of Rsrc1-161aa in energy metabolism. The total ATP level measurement assay showed elevated levels of ATP in *Rsrc1*^+*/*+^*/circRsrc1*^*−/−*^ cells than in *Rsrc1*^+*/*+^*/circRsrc1*^+*/*+^ cells, which could be rescued by overexpression of Rsrc1-161aa (Fig. 4a). We further analyzed oxygen consumption rate (OCR) to evaluate the level of OXPHOS in mitochondria, and extracellular acidification rate (ECAR) to study the change in glycolysis and lactate production in the cytoplasm using an XF-96 extracellular flux analyzer. As shown in Fig. 4b, basal and maximal OCRs were significantly decreased in *Rsrc1*^+*/*+^*/circRsrc1*^*−/−*^ cells. Concerning glycolysis, basal ECAR was significantly increased, indicating increased glycolysis activity in *Rsrc1*^+*/*+^*/circRsrc1*^*−/−*^ cells (Fig. 4c). Interestingly, overexpression of Rsrc1-161aa partially rescued the decrease in basal and maximal OCR levels in *Rsrc1*^+*/*+^*/circRsrc1*^*−/−*^ cells (Fig. 4b,c).

Since the loss of Rsrc1-161aa affects mitochondrial function, and most of the reactive oxygen species (ROS) are generated in mitochondria [31], we next measured the levels of ROS in *Rsrc1*^+*/*+^*/circRsrc1*^*−/−*^ cells. Dihydroethidium (DHE) staining revealed an increase in ROS levels in *Rsrc1*^+*/*+^*/circRsrc1*^*−/−*^ cells, which could be rescued by overexpression of Rsrc1-161aa (Additional file 1: Fig. S6a-b). ROS can induce DNA damage [32], thereby activating checkpoints and preventing the cell cycle [33]. We examined the protein levels of cyclin-dependent kinase 1 (Cdk1), which specifically regulates the G2/M phase transition [34]. Western blotting showed significant inhibition of Cdk1 activity, which was rescued by Rsrc1-161aa overexpression (Additional file 1: Fig. S6c). Overall, these results indicated that Rsrc1-161aa ensures the normal function of mitochondria, reduces the overproduction of ROS, and promotes cell cycle progression.

Since *Rsrc1*^+*/*+^*/circRsrc1*^*−/−*^ cells suffered from reduced OXPHOS, increased glycolysis, and increased ATP levels, we suspected that dysfunctional energy metabolism in *Rsrc1*^+*/*+^*/circRsrc1*^*−/−*^ sperm could be the reason for reduced motility. Notably, the ATP levels significantly increased in *Rsrc1*^+*/*+^*/circRsrc1*^*−/−*^ sperm than in control sperm (Fig. 4d). We further analyzed the OCR of *Rsrc1*^+*/*+^*/circRsrc1*^*−/−*^ sperm and found a significant decline in basal and maximal OCRs (Fig. 4e). Also, consistent with *Rsrc1*^+*/*+^*/circRsrc1*^*−/−*^ cells, mitochondrial glycolysis increased in *Rsrc1*^+*/*+^*/circRsrc1*^*−/−*^ sperm (Fig. 4f).

During glycolysis in sperm, lactate dehydrogenase C (LDHC), a testis-specific isozyme of LDH, catalyzes the conversion of pyruvate to lactate. Notably, pyruvate dehydrogenase (PDH) is the rate-limiting enzyme of OXPHOS [35]. Accordingly, we measured the change in activities of LDH and PDH in *Rsrc1*^+*/*+^*/circRsrc1*^*−/−*^ cells and sperm. We found an increase in LDH activity but a decrease in PDH activity, both of which were evidently rescued by the overexpression of Rsrc1-161aa in *Rsrc1*^+*/*+^*/circRsrc1*^*−/−*^ cells (Fig. 4g,h). The same was true in knockout sperm (Fig. 4i–k). Further measurement of glycolysis products in *Rsrc1*^+*/*+^*/circRsrc1*^*−/−*^ sperm showed a significant increase in basal lactate excretion rate and lactate level (Fig. 4l,m); this was consistent with increased glycolysis activity. Overall, *Rsrc1*^+*/*+^*/circRsrc1*^*−/−*^ sperm exhibited an imbalance of mitochondrial metabolism, a decrease in OXPHOS, and an increase in glycolysis, which raised total ATP production and lactate level.

### Rsrc1-161aa regulates the translation of OXPHOS proteins

**Fig. 5:**
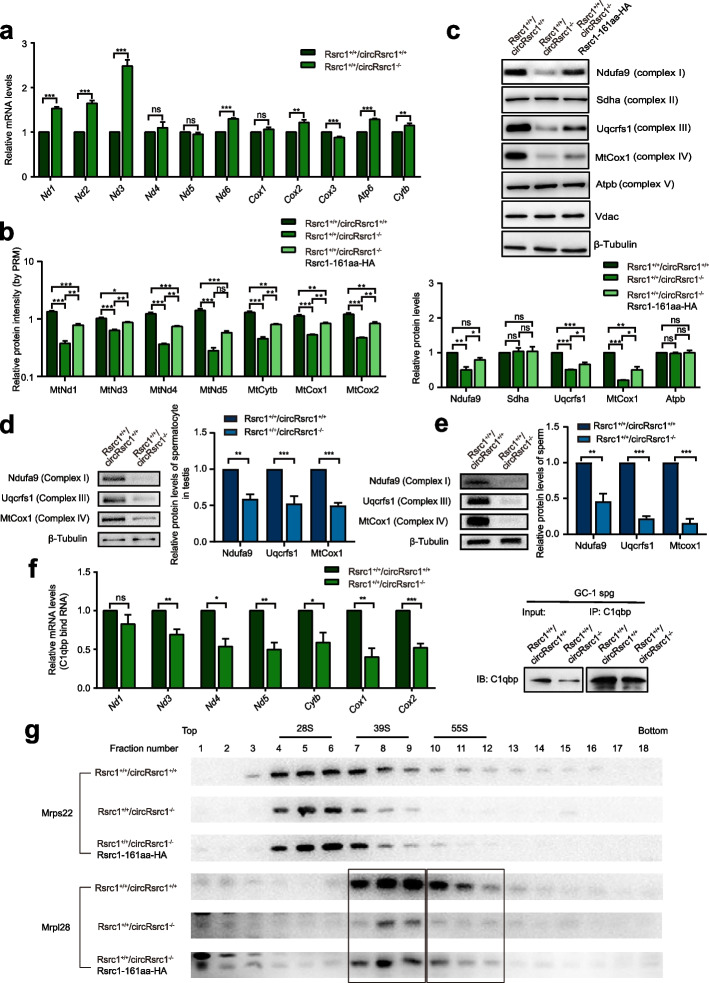
Rsrc1-161aa regulates mitochondrial ribosome assembly and protein translation. **a** RT-qPCR detection of mitochondrial ribosomal gene transcript levels in wild-type and *Rsrc1*^+*/*+^*/circRsrc1*^*−/−*^ cells. RNA data were normalized by 18S (*n* = 3). **b** Mass spectrometry targeted quantitative detection of proteins encoded by mitochondria in wild-type, *Rsrc1*^+*/*+^*/circRsrc1*^*−/−*^ and rescue cells (*n* = 3). **c** Western blotting of mitochondrial respiratory proteins in wild-type, *Rsrc1*^+*/*+^*/circRsrc1*^*−/−*^ and rescue cells. The statistical graph shows the gray values after three repetitions (*n* = 3). **d** Flow cytometry was used to separate spermatocytes from testis, and Western blotting was performed to detect mitochondrial respiratory proteins. The statistical graph shown the gray values after three repetitions. **e** Western blotting of mitochondrial respiratory protein in mouse sperm. The statistical graph shows the gray values after three repetitions (*n* = 3). **f** RIP-qPCR experiment in wild-type and *Rsrc1*^+*/*+^*/circRsrc1*^*−/−*^ cells using the anti-C1qbp antibody (*n* = 3). **g** Cell lysates from wild-type, *Rsrc1*^+*/*+^*/circRsrc1*^*−/*^.^*−*^ and rescue cells were separated by 15 ~ 30% sucrose density gradient and analyzed using antibodies against Mrps22 (28S small subunit) and Mrpl28 (39S large subunit) proteins. All data are means ± SEM. When performing statistical tests between two groups of data, tested using a two-tailed unpaired *t*-test. When performing statistical tests between three or more groups of data, tested using one-way ANOVA with Tukey’s post hoc analysis. *P < 0.05; **P < 0.01; ***P < 0.001

To investigate the molecular mechanism of reduced OXPHOS activity in *Rsrc1*^+*/*+^*/circRsrc1*^*−/−*^ cells, we measured the amount of mitochondrial DNA by qPCR and found no significant change (Additional file 1: Fig. S7a). Moreover, mRNA level analysis of mitochondrial genes from five multi-subunit enzymatic complexes showed upregulation or no significant change in *Rsrc1*^+*/*+^*/circRsrc1*^*−/−*^ cells (Fig. 5a). We then quantified the expression levels of 7 mtDNA-encoded OXPHOS proteins using PRM-based MS targeted quantification. The expression of all the quantified proteins was downregulated. Importantly, overexpression of Rsrc1-161aa partially rescued the downregulated expression of 6 of the 7 mtDNA-encoded proteins in Rsrc1^+/+^/circRsrc1^*−*/*−*^ cells (Fig. 5b). These results suggest that Rsrc1-161aa might be involved in translational regulation of mtDNA-encoded OXPHOS proteins. Furthermore, Western blotting confirmed the significantly reduced levels of mtDNA-encoded COX1, a subunit of complex IV, in *Rsrc1*^+*/*+^*/circRsrc1*^*−/−*^ cells. Also, the protein levels of nuclear-encoded Ndufa9 (complex I) and Uqcrfs1 (complex III) were downregulated despite no change in their transcript levels in Rsrc1^+/+^/circRsrc1^−/−^ cells (Fig. 5c, Additional file 1: Fig. S7b). Such changes could have destabilized the mitochondrial complex. Meanwhile, we also estimated the levels of these differential OXPHOS proteins in vivo. As shown in Fig. 5d and e, Ndufa9, Uqcrfs1, and Cox1 were all downregulated in Rsrc1^+/+^/circRsrc1^*−*/*−*^ spermatocyte and sperm.

Previous studies showed that C1qbp interacts with mitochondrial ribosomal proteins and thereby regulates mtDNA-related protein synthesis [29]. To analyze whether the absence of Rsrc1-161aa in *Rsrc1*^+*/*+^*/circRsrc1*^*−/−*^ cells affected interaction between C1qbp and mitochondrial ribosomal proteins, we performed co-immunoprecipitation of C1qbp followed by Western blotting. The results showed that interaction between C1qbp and mitochondrial ribosomal proteins Mrps22, Mrps29, and Mrpl3 remained unaffected in *Rsrc1*^+*/*+^*/circRsrc1*^*−/−*^ cells (Additional file 1: Fig. S7c). We then examined the expression level of these mitochondrial ribosomal proteins and found no change (Additional file 1: Fig. S7d). Therefore, we concluded that Rsrc1-161aa does not regulate the interaction between C1qbp and mitochondrial ribosomal proteins.

Interestingly, C1qbp can also bind to mitochondrial RNA and may regulate mitochondrial protein synthesis [29]. It could be that Rsrc1-161aa, as a C1qbp-interacting protein, might regulate mitochondrial translation by affecting C1qbp binding to mitochondrial mRNA. Accordingly, we analyzed the changes in mitochondrial RNA-binding activity of C1qbp using RNA immunoprecipitation (RIP) followed by RT-qPCR. The results showed that C1qbp binding to 6 of the 7 mitochondrial transcripts was significantly decreased in *Rsrc1*^+*/*+^*/circRsrc1*^*−/−*^ cells (Fig. 5f). This suggested that the deletion of Rsrc1-161aa altered the mitochondrial mRNA binding ability of C1qbp. To study whether Rsrc-161aa affects the function of mitochondrial ribosomes, we performed sucrose gradient profiling of mitochondrial ribosomes. The small and large mitochondrial ribosomal subunits were detected by antibodies against Mrps22 and Mrpl28, respectively. We found that the proportions of large ribosomal subunit and monosome were significantly decreased in *Rsrc1*^+*/*+^*/circRsrc1*^*−/−*^ cells, while small ribosomal subunit showed a mild decrease. Interestingly, these phenotypes could be rescued by overexpression of Rsrc1-161aa (Fig. 5g). Taken together, Rsrc1-161aa regulates the mitochondrial mRNA binding ability of C1qbp, which affects the formation of the large mitochondrial ribosomal subunit and monosomes ensuring the normal synthesis of mitochondrial proteins such as OXPHOS proteins.

## Discussion

Previous studies showed that circRNAs can act as molecular chaperones facilitating the mitochondrial entry of nuclear-encoded proteins [36]. In this study, we found that *circRsrc1* encoded Rsrc1-161aa which interacts with C1qbp and regulates the assembly of mitochondrial ribosomes and translation of OXPHOS proteins. Also, Rsrc1-161aa regulates the energy metabolism in germ cells and is important for sperm generation, sperm motility, and male fertility.

We found that the deletion of *circRsrc1*^*322bp*^ composed of exons 2 and 3 resulted in a compensatory activation of another circular RNA *circRsrc1*^*496bp*^ composed of exons 2, 3, and 4 in mouse testis and in vitro GC-1 spg cell line. circRNAs are produced by back-splicing and by joining of downstream splice donor site to an upstream splice acceptor site [37]. A single gene can produce multiple circRNAs, which is regulated by competition for RNA pairing across flanking introns [28]. Since there was no phenotype difference between *Rsrc1*^+*/*+^*/circRsrc1*^*−/−*^ and *Rsrc1*^*−/−*^*/circRsrc1*^*−/−*^ mice, the activated *circRsrc1*^*496bp*^ could be non-functional in spermatogenesis. However, whether it regulates the production of *circRsrc1*^*322bp*^ remains to be explored. To get accurate conclusions, the examination of possible activation of other spliced forms of circRNAs from the same gene is important for circRNA knockout studies.

Here, we showed that *circRsrc1* encoded Rsrc1-161aa which interacts with C1qbp and regulates mitochondrial ribosome assembly. C1qbp is an evolutionarily conserved protein involved in mitochondrial protein synthesis [29, 30]. Mam33, a yeast homolog of C1qbp, is necessary for the proper assembly of the large mitochondrial ribosome subunit in yeast [38]. C1qbp deficiency in mice affects the formation of functional 55S mitochondrial ribosomes [29]. However, how C1qbp itself is regulated in mitochondrial ribosome assembly and translation is unknown. Here, we observed impaired translation of OXPHOS mitochondrial proteins in *Rsrc1*^+*/*+^*/circRsrc1*^*−/−*^ cell line and *Rsrc1*^+*/*+^*/circRsrc1*^*−/−*^ mouse sperm. Meanwhile, the formation of large ribosomal subunit and 55S monosome was affected in *Rsrc1*^+*/*+^*/circRsrc1*^*−/−*^ cell line. Thus, we concluded that Rsrc1-161aa encoded by *circRsrc1* can be an important regulator of mitochondrial ribosome assembly through interacting with C1qbp.

The analysis of *Rsrc1*^+*/*+^*/circRsrc1*^*−/−*^ spermatocytes showed decreased levels of some OXPHOS proteins and meiotic arrest. In the testis, spermatocytes utilize lactate from Sertoli cells as the main energy source [39]. Lactate is oxidized to pyruvate, which is then transported into the mitochondria and oxidized by pyruvate dehydrogenase (PDH) complex to acetyl-CoA for subsequent oxidative phosphorylation [40–42]. The deletion of PDHA2 causes meiotic arrest [41]. During spermatogenesis, spermatocytes undergo an acute upregulation of OXPHOS [40]. Given the critical role of OXPHOS in spermatocytes, dysfunction of OXPHOS in *Rsrc1*^+*/*+^*/circRsrc1*^*−/−*^ spermatocytes is expected to cause meiotic arrest.

In this study, *Rsrc1*^+*/*+^*/circRsrc1*^*−/−*^ sperm had reduced OXPHOS, increased ATP level, and decreased sperm motility. In sperm, ATP is generated in the compartmentalized metabolic pathways via glycolysis and OXPHOS; glycolysis occurs in the principal piece while OXPHOS occurs in mitochondria in the mid-piece of flagellum [43]. There is a direct and positive correlation between sperm motility and all mitochondrial respiratory chain complex activities [44]. A previous report also showed a decrease in sperm OXPHOS protein levels in asthenozoospermia [45]. However, even with impaired OXPHOS, *Rsrc1*^+*/*+^*/circRsrc1*^*−/−*^ mouse sperm had elevated ATP levels. Since part of the energy for sperm motility comes from glycolysis [46–48], sperm in *Rsrc1*^+*/*+^*/circRsrc1*^*−/−*^ mice may have experienced a compensatory increase in glycolysis, which led to an increase in sperm lactate levels. Previous studies have shown that high lactate levels help bird sperm remain stationary in the sperm storage tubules [49]. On the same line, a decrease in pH was shown to reduce sperm motility in human semen [50]. In summary, both a decrease in OXPHOS levels and an increase in lactate levels may be important for reducing sperm motility in *Rsrc1*^+*/*+^*/circRsrc1*^*−/−*^ mice.

## Conclusions

In summary, we identified a novel protein Rsrc1-161aa encoded by *circRsrc1*, which plays important regulatory roles in spermatogenesis and male fertility. Rsrc1-161aa along with C1qbp may also regulate the assembly of ribosomes and translation of OXPHOS proteins in mitochondria (Additional file 1: Fig. S7e). In general, circRNAs may encode proteins with extensive physiological regulatory functions in mammals.

## Methods

### Animals

C57/BL6 mice used in the present study were housed in a specific pathogen-free (SPF) environment with free water and food access, followed the circadian rhythm of the mice. We conducted animal breeding and experiments according to the requirements of the Institutional Animal Care and Use Committee of Nanjing Medical University (Approval No. IACUC-1812007).

### Bioinformatic analyses

First, the sequencing data (paired-end, 101 bp) of two replicate experiments of mouse testes were combined. Fastp is an ultra-fast FASTQ format file processing software that integrates quality control, filtering, correction, preprocessing, and other functions. Fastp (0.18.0) is used to submerge merged reads and filter and trim the quality of reads under the default parameters [51]. Subsequently, in order to exclude the interference of rRNA, SortmeRNA [52] was used to align and remove all rRNA sequences on the mouse genome, filtering a total of about 62.42% reads. CIRI2, CIRCexplorer2, and find_circ were mainly used in the identification of circRNAs. For CIRI2, reads need to be aligned to the genome before identification. Here, BWA-MEM algorithm and -T 20 parameter were used for alignment. In the identification of CIRCexplorer2, the parameters of Tophat2 and –fusion-search –keep-fasta-Order –no-coverage-search – Bowtie1 were used for comparison. Find_circ used bowtie2 and –very sensitive –mm –score-min = C,-15,0 parameters for comparison and extracted unmatched reads and used its find_circ.py script for identification of reverse splicing reads. The data can be found in Additional file 2: Table S1.

### Targeted protein quantification by parallel reaction monitoring

The samples were lysed in lysis buffer consisting of 8 M Urea (Sigma, U6504), 10 mM sodium pyrophosphate (Sigma, S6422), 1 mM NaF (Supelco, 1,064,490,250), 1 mM sodium orthovanadate (Sigma, 450,243), 1 mM β-glycerophosphate, 1% EDTA-free protease inhibitor, 75 mM NaCl (Sigma), and 50 mM Tris (Sigma), pH 8.2), reduced by DTT (Thermo Fisher Scientific, 20,290) and alkylated by IAA (Sigma). Protein digestion was performed using 5 ng/μl Trypsin (Promega) for 16 h at 37 °C. The peptides were desalted by the C18 column (Thermo Fisher Scientific) and dried before use.

The crude isotope-labeled heavy synthetic peptides (Additional file 3: Table S2) were purchased from Synpeptide. To quantify other proteins than circRsrc1, each 500 ng peptide sample was combined with the heavy synthetic peptides for PRM quantification. To quantify circRsrc1 protein in GC-1 spg cells or mouse testes, 120 μg peptide samples were combined with the heavy synthetic peptide and pre-fractionated by an xBridge BEH130 C18 column (1 mm × 150 mm, 1.7 μm, Waters) using the M-class HPLC system (Waters). Sixty fractions were collected in one fraction per minute with a 140-min gradient and were subjected to the following targeted quantification by PRM.

For PRM quantification, the peptide samples containing heavy synthetic peptides were separated by an analytical column (75um, 1.7 μm × 15 cm, USA) using a flow rate of 300 nL/min on an easy-nLC 1200 HPLC system (Thermo Fisher Scientific) in a 30-min (3% to 5% buffer B for 3 s, 5 to 15% buffer B for 11 min and 56 s, 15 to 28% buffer B for 10 min 30 s, 28 to 38% buffer B for 3 min and 45 s, 38 to 100% buffer B for 3 s, and 100% buffer B for 3 min and 43 s) or 60-min gradient (3–5% B for 5 s, 5–15% B for 23 min and 55 s, 15–28% B for 21 min, 28–38% B for 7 min and 30 s, 38–100% B for 5 s, and 100% B for 7 min and 25 s). Analysis was conducted using a scheduled method on the Orbitrap Fusion™ Lumos™ mass spectrometer with the following parameters: a higher-energy collision of 30 eV, an AGC target of 5E4, a maximal injection time of 118 ms, and a scan range (m/z) of 150–2000.

PRM data were processed with Skyline Daily software. At least three transitions per precursor were used to quantify the targeted peptides in the samples. For relative quantification, the protein expression was calculated as the ratio of the transition peak areas of endogenous to those of the heavy synthetic peptide.

### RNA extraction, RT-PCR, and RT-qPCR

Total RNA was extracted from indicated samples using Trizol reagent (Thermo Fisher Scientific, 15,596,026). Total RNA was reversely transcribed into cDNA using PrimeScript™ RT reagent Kit (Takara, RR037A) with random hexamers according to manufacturer protocols. Then, the cDNA was used for RT-PCR with 2 × Rapid Taq Master Mix (Vazyme, P515) or RT-qPCR analysis with AceQ qPCR SYBR Green Master Mix (Vazyme, Q131). All reactions were run in triplicate. The relative quantification values were calculated by the 2^−ΔΔCt^ method using 18S or GADPH as an internal reference. To avoid unspecific amplification of linear RNAs, divergent primers were designed to specifically detect circRNAs. The sequences for the primers can be found in Additional file 3: Table S3.

### Western blotting and silver staining

The cell protein was extracted by 8 M buffer (8 M Urea, 10 mM sodium pyrophosphate, 1 mM NaF, 1 mM sodium orthovanadate, 1 mM β-glycerophosphate, 1% EDTA-free protease inhibitor, 75 mM NaCl, 50 mM Tris, pH 8.2) and subsequently quantified protein concentration with Bradford Protein Assay Kit (Beyotime, P0006). The sperm were lysed in lysis buffer (100 mM Tris–HCl pH 8.8, 8‰ bromophenol blue, 20% glycerol, 20 mM EDTA, 200 mM DTT, 4% SDS) by sonication. Equivalent protein lysates were separated on 12% SDS-PAGE gel and then transferred to polyvinylidene difluoride membranes. Followed blocked with 5% skimmed milk in TBS at room temperature for 2 h, the diluted primary antibodies were incubated at 4° overnight. After washing the membranes with TBST every 15 min for 4 times, diluted horseradish peroxidase (HRP)-conjugated secondary antibodies were hybridized with the membranes for 2 h at room temperature. Finally, the signals were detected by chemiluminescence solution ECL (Vazyme, E412). ImageJ software was used to quantify western blotting bands. Antibodies and dilution concentrations are shown in Additional file 3: Table S4. For silver staining, gels were stained using the Fast Silver Stain Kit (Beyotime, P0017S) as the protocol described. Afterwards, differential gel bands were manually excised and detected by mass spectrometry.

### Cell cultures, plasmids, and transfection

Spermatogonium GC-1 spg cells, spermatocyte GC-2spd(ts) cells, and mouse Neuro-2a (N2a) neuroblastoma cells were obtained from American Type Culture Collection (ATCC) and were cultured in Dulbecco’s modified Eagle’s medium supplemented with 10% fetal bovine serum (Gibco, 16,000), 1% penicillin and streptomycin (Gibco, 15,140–122) at 37 °C under 5% CO_2_.

For the artificial ATG mutant plasmid (circRsrc1-ATGmut), the full-length *circRsrc1* sequence was amplified from cDNA, in which the start codon (ATG) of *circRsrc1* was replaced with CTG. The mutated *circRsrc1* sequence containing appropriate overhangs to recombine with the linearized backbone was then cloned into the vector with side-flanking acceptor and donor sequences from laccase2 gene by using the ClonExpress One Step Cloning Kit (Vazyme, C113). The empty vector containing only side-flanking acceptor and donor sequences from laccase2 gene was used as a negative control.

The Rsrc1-161aa-HA-IRES-eGFP plasmid was constructed by using PB vector. Briefly, the full-length linearized Rsrc1-161aa coding sequence was amplified by donor cDNA. At the same time, we inserted an HA and eGFP epitope at the C-terminus of the sequence and ensure that it contains appropriate overhangs to recombine with the linearized backbone. Then, we cloned the sequence into PB vector by using the ClonExpress One Step Cloning Kit (Vazyme, C113). The Rsrc1-161aa-eGFP plasmid containing linearized Rsrc1-161aa coding sequence with an eGFP epitope at the C-terminus and appropriate overhangs. Then, it was cloned into the pcDNA3.1 (Invitrogen) vector by using the ClonExpress One Step Cloning Kit (Vazyme, C113). The C1qbp-HA was constructed by using the pcDNA3.1 backbone. In brief, the full-length cDNA sequence of *C1qbp* was amplified from donor cDNA, and an HA epitope was inserted into the C-terminal of C1qbp cDNA. Then, we cloned the sequence into pcDNA3.1 vector using the ClonExpress One Step Cloning Kit (Vazyme, C113). E. coli DH5α was used for cloning of plasmids and correct plasmid construction was confirmed by Sanger sequencing.

Transfection was carried out using Lipofectamine 2000 (Thermo Fisher Scientific, 11,668,030) according to manufacturer instructions. After transfection for 48 h, cells were collected for the further experiments.

### Generation of knockout mice, knockout cell line, and stable rescue cell line

To knock out the parental gene Rsrc1, we designed guide RNAs (gRNA A: 5′-CCTCGTCCACGCTCACATAGTAG-3′ and gRNA B: 5′-GTCACAGAAGAACCCGTAGCAGG-3′) onto exon4 of the Rsrc1 gene (NC_000069). We designed guide RNAs (gRNA A: 5′-GCTGTGTCCAAACTCCTCCCAGG-3′ and gRNA B: 5′-TCAATTGCATTTTAAATTCAAGG-3′) onto intron between exon3 and exon4 of the Rsrc1 gene to knock out the circRsrc1 rather than parental gene. We designed guide RNA (5′-CCAGAAGGTATGTGCAGGAGTAC-3′) of the Rsrc1 to knock out the circRsrc1 and parental gene. By in vitro transcription, we obtained Cas9 mRNA and gRNA, and then both were co-injected into the cytoplasm of single-cell C57BL/6 J embryos. The injected embryos were transferred into the uteri of recipient pseudo pregnant females. Genotypes of the mice were identified by PCR amplification followed by Sanger sequencing.

To knockout circRsrc1 in GC-1 spg cell line, two gRNAs (gRNA A: 5′-GCTGTGTCCAAACTCCTCCCAGG-3′ and gRNA B: 5′-GCTGCAGATGGCTTGCGTCAGGG-3′) that specifically target intron between exon3 and exon4 of the Rsrc1 gene were designed and be cloned into plasmid pPGL3-U6-PGK-sgRNA-puromycin. Plasmids expressing Cas9 and sgRNAs were co-transfected to the GC-1 spg cells. Twenty-four hours after transfection, 2 μg/ml puromycin (Thermo Fisher Scientific, A1113803) and 10 μg/ml Blasticidin (Thermo Fisher Scientific, A1113903) were added intracellularly for 48 h. Some viable cells were harvested for DNA extraction using the QuickExtract DNA Extraction Solution (Epicentre, QE09050) for DNA extraction. The T7 endonuclease (NEB, M0302L) were used to detect the efficiency of indels. Using the flow cytometer (FACSAria Fusion SOP), residual cells were seeded to 96-well plates at a density of single cell per dish. PCR and RT-PCR were performed after single-cell clone amplification, and Sanger sequencing was used to confirm that circRsrc1 was knocked out and the parental gene was not affected. The Rsrc1-161aa-HA-IRES-eGFP was used for stable Rsrc1-161aa expression. The knockout cell lines were transfected using Lipofectamine 2000 (Thermo Fisher Scientific, 11,668,030), followed by GFP sorting with flow cytometry for 1 mouth to obtain stable rescue cell lines.

### Subcellular fractionation

The Cytoplasmic and Nuclear RNA Purification Kit (Norgen Biotek, 21,000) was used to isolate cytoplasmic and nuclear RNA according to the manufacturer’s protocols. The RNAs were then reverse transcribed into cDNA using the PrimeScript™ RT reagent Kit (Takara, RR037A) and random hexamers. 18S and U6 served as the cytoplasmic and nuclear controls, respectively.

### Fertility test

The fertility test was carried out by mating each male sexually mature mice with two wild-type C57/BL6 females for a minimum of 2 months, with each knockout male mice subjected to the same feeding conditions as controls. All the mice used for the fertility test were 8 ~ 12 weeks old. The number of pups was recorded.

### Epididymal sperm motility analysis

The epididymis was dissected from adult mice. The sperm were squeezed from the caudal epididymis and incubated in modified HTF medium (Irvine Scientific, 90,126) containing 10% fetal bovine serum at 37 °C under 5% CO_2_. After incubation for 10 min, the mouse sperm were analyzed by computer aided semen analysis detection (Hamilton Thorne Research Inc.). Motility was scored for at least 200 cells per sample.

### Histology hematoxylin and eosin (H&E) staining and TUNEL assay

Testicular and epididymal tissues from male mice were placed in modified Davidson’s fluid for 24 h, dehydrated in gradient ethanol, embedded in paraffin, and sectioned at 5 μm. The sections were deparaffinfized and rehydrated for histological examination with HE. Sperm was spread on a glass slide and be fixed in 4% PFA at room temperature for 30 min, and then HE staining and morphological observation were performed. For TUNEL assays, TUNEL Bright Green Apoptosis Detection Kit (Vazyme, A113) was used according to manufacturer instructions.

### Fluorescence microscopy

Cells were grown on chamber slides precoated with fibronectin (Sigma, FC010). The cells were washed 3 times with phosphate buffer saline (PBS) and then fixed with 4% paraformaldehyde for 15 min; after three times washes with PBS, the samples were blocked with 1% BSA in PBS containing 0.1% Triton X-100(PBS-T) and then incubated with primary antibodies at 4 °C overnight. The next day, after incubation with corresponding secondary antibody and Hoechst 33,342 at room temperature for 2 h, the samples were washed in PBS. Cells were loaded with 200 nM Mitotracker (Beyotime, C1035) for 30 min in a humidified incubator with 5% CO_2_ at 37 °C. After fixation, permeabilization, and blocking, cells were then incubated with anti-C1qbp overnight at 4 °C. DHE (Vigorous Biotechnology Beijing Co, Ltd, R001) was used to detect intracellular superoxide. DHE (5 μM) was added to cells and incubated at 37 °C for 90 min in the dark. At the end of the incubation time, cells were washed with PBS. Images were captured under the Zeiss LSM800 confocal microscope. Use ImageJ software to analyze cells.

### Flow cytometry

The seminiferous tubules of the mouse testes were washed with 1 × PBS, and then digested and filtered with collagenase and trypsin to obtain a single-cell suspension. Cells were stained with Hoechst to distinguish 1C, 2C, and 4C based on their DNA content.

### *RNA fluorescent *in situ* hybridization (FISH)*

The RNA fluorescence in situ hybridization (FISH) probe targeting *circRsrc1* were designed and produced by GenePharma (shanghai, China) using oligonucleotide probes with high specificity. To eliminate interference from *Rsrc1*, we selected nearly equal-length sequences on both sides of the junction site as the probe sequence (5′- GCGTCCCATTTCTTCTGGTTCTG-3′) for specific detection of *circRsrc1*. The localization of *circRsrc1* was measured using the FISH kit (GenePharma, #F03301), following manufacturer protocols. The Zesis LSM800 confocal microscope was used to view the immunofluorescence of each slide.

### Cell cycle analysis

For cell cycle assay, cells were harvested and washed with cold PBS for two times, then fixed in cold 70% ethanol in PBS overnight at − 20 °C. The fixed cells were centrifuged at 1500 rpm for 10 min, then washed with PBS twice to remove ethanol. PI/rNase staining solution (BD Pharmingen™, 550,825) was then added into the cells, followed by incubation at room temperature for 15 min. Finally, the cells were analyzed via flow cytometry.

### CCK-8 assay

For CCK-8 assays, 3500 cells were seeded into each well of a 96-well plate. The viability of cells was determined at 24, 48, 72, and 96 h using a CCK-8 Cell Counting Kit (Vazyme, A311) by measuring the absorbance value at the 450 nm following manufacturer instructions.

### Co-immunoprecipitation

Transiently transfected cells were collected and washed 3 times with PBS, then lysed with 1 ml of RIPA buffer (Beyotime, P0013C) for 40 min at 4 °C. After centrifugation at 13,000* g* for 30 min, the supernatant was collected and pre-cleared with 30 µl protein A/G beads (Bimake, B23202) for 1 h at 4 °C. The pre-cleared lysates were collected and incubated with indicated antibodies overnight at 4 °C. The protein complexes were added into 60 μl of protein A/G magnetic beads for 4 h at 4 °C, then the supernatant was removed and the beads were washed 5 times with RIPA buffer. Next, SDS protein loading buffer was added, and after boiled at 95 °C for 10 min, the collected denatured protein complexes were separated via SDS-PAGE and probed with the indicated antibodies. Rabbit Normal IgG was used as a control for co-immunoprecipitation.

### Proximity ligation assay (PLA)

PLA was performed according to manufacturer recommendations of Duolink® In Situ Orange Starter Kit Mouse/Rabbit (Sigma, DUO92102). The transiently transfected cells were collected and fixed with 4% paraformaldehyde for 15 min at room temperature. After blocked with the blocking solution, cells were incubated with mouse anti-HA and rabbit anti-C1qbp overnight at 4 °C with no primary antibody added as a negative control. After incubation with PLA probes, ligation and amplification are performed. Cells were analyzed with a × 60 objective on a Zeiss LSM800 confocal microscope.

### ATP measurements

To detect the ATP content of cell, 5000 cells were seeded into each well of 6-well plates. After a certain period of culture, cells were harvested and washed with PBS twice. To detect the ATP content of sperm, sperm were collected after incubation in medium at 37 °C under 5% CO_2_. Then, the ATP levels of samples were detected by using ATP assay kit (Beyotime, S0026) according to the manufacturer instructions.

### Metabolic analysis

The real-time measurement of oxygen consumption rate (OCR) and extracellular acidification rate (ECAR) of cells were analyzed using a Seahorse XF96 extracellular flux analyzer (Seahorse Biosciences, North Billerica, MA). In short, 10,000 cells were planted in each well and cultured for a certain period. OCR of the cells was obtained by sequential addition of oligomycin (1 μM), FCCP (1 μM), and antimycin A (2 μM) plus rotenone (1 μM). We measured ECAR during sequential injection of glucose (10 mM), oligomycin (1 μM), and 2-DG (100 mM). The OCR and ECAR values were normalized by cell counts.

As previously described [53], an XF96 extracellular flux analyzer was used to measure the real-time OCR of sperm. Briefly, 1.5 × 10^5^ sperm cells were seeded on each well of a XF96 plastic microplate which had been previously coated with 0.5 mg/ml concanavalin A (Sigma, L7647). After standing for 1 min, the plate was centrifuged for 2 min at 800* g*. Then, 1 μM FCCP and 1 μM rotenone plus 1 μM antimycin A were added in sequence to detect OCR. The OCR values were normalized by sperm count. For Extracellular pH analysis of sperm, a fluorescence kit (Abcam, ab197244) was used following manufacturer protocols. Each measurement was normalized by number of the sperm.

### Pyruvate dehydrogenase activity, lactate dehydrogenase C content and activity detection

For pyruvate dehydrogenase activity analysis, an ELISA kit was used (YIFEIXUE BIO TECH, YFX0135) following manufacturer protocols. Lactate dehydrogenase activity in cells was measured using the Mouse LDH ELISA kit (YIFEIXUE BIO TECH, YFXEM00024-2). For sperm Lactate dehydrogenase C content and activity analysis, ELISA kits were used (YIFEIXUE BIO TECH, YFXEM00072-1 and YIFEIXUE BIO TECH, YFXEM00072-2) following manufacturer protocols. Values were normalized for protein content obtained.

### Sperm lactate and lactate excretion rate

For sperm lactate and lactate excretion rate measurement, the caudal epididymis was placed in modified Tyrode’s medium [53] and incubated at 37 °C under 5% CO_2_. An aliquot of sperm was collected at 0, 60, 120 min. After centrifugation at 300* g* for 10 min, the sediment and supernatant were frozen in liquid N_2_. Lactate levels in the sperm and the supernatant were determined using a commercial kit (BioVision, K607), following manufacturer protocols.

### Measurement of mtDNA copy numbers

To measure the mtDNA copy number, genome DNA was extracted from each cell line, followed by qPCR analysis. The relative mtDNA copy number was confirmed based on the ratio of mtDNA/nuclear (n)DNA (CYTB/AT-III).

### RNA-IP

RNA immunoprecipitation (RIP) was carried out using an EZ-Magna RIP Kit (Millipore, 17–701) according to manufacturer instructions. Briefly, C1qbp antibody was first bound to 50 µl protein A/G beads for 1 h at room temperature, followed by binding to triplicate samples of cells lysates at 4 °C for 12 h. Then, protein was digested with proteinase K and 10% SDS, followed by RNA extraction from samples. The eluted RNA was quantitated using the Qubit™ RNA high sensitivity (HS), broad range (BR) and extended range (XR) Assay Kits (Invitrogen, Q32852) and measured on the Qubit 4.0 Fluorometer (Invitrogen).

### Analysis of mitochondrial ribosomes on sucrose gradients

Cells were lysed in buffer (100 mM KCl, 2 mM MgCl_2_, 10% glycerol, 50 mM HEPES, 150 mM NaCl, 1 mM DTT, 0.1% Triton) with 1 × proteinase inhibitor cocktail without EDTA. Total cell lysates were loaded onto a linear 10–30% sucrose gradient (gradient buffer: 10 mM Tris–HCl pH 7.2, 100 mM NaCl, 5 mM MgCl_2_, 1 mM DTT) and centrifuged at 36,000 rpm for 3 h at 4 °C. After separation, proteins were precipitated with methanol and resolved by SDS-PAGE.

### Statistical analysis

Analysis of variance (ANOVA) and Student’s *t* test were used to compare the differences with controls. All experiments were repeated at least three times. Statistical significance was accepted if *p* < 0.05.

## Supplementary Information

**Additional file 1: Fig. S1.** Peptides translated by detected circRNAs are present in mouse testes. a. The specific peptides translated by circRNAs were detected by mass spectrometry and the corresponding spectra are shown. Fig. S2. The detected circRNAs are present in mouse testes. a. Linear splicing forms of the gene of interest were detected specifically in mouse testes by convergent primers. Both divergent primers detected circular forms in cDNA but not in gDNA of mouse testes. Sanger sequencing confirmed the presence of circular RNAs. Fig. S3. Different knockout mice were constructed using the CRISPR/Cas9 system. a. Relative expression levels of Rsrc1 in mouse testes at different ages in weeks. b. Relative expression levels of Rsrc1 in different germ cells and somatic cells of mouse testes. c. Subcellular localization of circRsrc1 in mouse testes was determined by RT-qPCR. 18S and U6 were used as internal controls. d. Schematic of the targeted exon 4 of mouse and sequence from wild-type and Rsrc1−/−/circRsrc1+/+ mouse. Sanger sequencing after PCR confirmed that the knockout was successful. e. Identification of Rsrc1−/−/circRsrc1+/+ mice by PCR. f. RT- PCR analysis of circRsrc1 and Rsrc1 mRNA levels in wild-type and Rsrc1−/−/circRsrc1+/+ mice. g. Western blotting of Rsrc1 protein in wild-type and Rsrc1−/−/circRsrc1+/+ mice. h. Schematic of the targeted intron of mouse and sequence from wild-type and Rsrc1+/+/circRsrc1−/− mouse. Sanger sequencing after PCR confirmed that the knockout was successful. i. Identification of Rsrc1+/+/circRsrc1−/− mice by PCR. j. RT-PCR analysis of circRsrc1 mRNA levels in wild-type and Rsrc1+/+/circRsrc1−/− mice. Sanger sequencing was performed after PCR. k. Schematic of the targeted intron and exon 3 of sequences from wild-type and Rsrc1−/−/circRsrc1−/− mouse. Sanger sequencing after PCR confirmed that the knockout was successful. l. RT-PCR analysis of circRsrc1 and Rsrc1 mRNA levels in wild-type and Rsrc1−/−/circRsrc1−/− mice. m. RT-qPCR analysis of circRsrc1 and Rsrc1 mRNA levels in wild-type and Rsrc1−/−/circRsrc1−/− mice. n. Western blotting of Rsrc1 protein in wild-type, Rsrc1+/+/circRsrc1−/− and Rsrc1−/−/circRsrc1−/− mice. Fig. S4. Phenotypic analysis of different knockout mice. a. The testicular/body weight ratio of different knockout and wild-type mice are shown. b. Percentage of TUNEL positive tubules in mouse testes. A TUNEL positive tubule contained at least one TUNEL positive cell. c. The average number of TUNEL positive cells in each tubule of mouse testes. d. H&E staining of the testis, caupt epididymidis, cauda epididymidis and sperm from adult wild-type and different knockout mice. Bar, 50 μm. All data are means ± SEM, tested using one-way ANOVA with Tukey’s post hoc analysis *P < 0.05; **P < 0.01; ***P < 0.001. Fig. S5. Construction of knockout cell line. a. RT-qPCR was performed using divergent primers to detect the expression of circRsrc1 in cDNA from GC-1 spg cells and GC-2spdcells. All data are means ± SEM, tested using a two-tailed unpaired t-test; ***P < 0.001. b. Detection of the circular form of circRsrc1 in cDNA of GC-1 spg cells by using divergent primers. Sanger sequencing was performed to confirm the junction site of circRsrc1. c. Mass spectrometry detection of peptides translated by circRsrc1 from wild-type and Rsrc1+/+/circRsrc1−/− cells. d. Establishment of Rsrc1+/+/circRsrc1−/− cell line using CRISPR/Cas9 technology. e. Validation of Rsrc1+/+/circRsrc1−/− cells by PCR. f. FISH with junction-specific and linear-specific probes for circRsrc1 and linear Rsrc1, respectively, in vitro. Bar, 10 μm. g. RT-PCR analysis of circRsrc1 mRNA levels in wild-type and Rsrc1+/+/circRsrc1−/− cells. Sanger sequencing was performed following PCR. h. Rsrc1-161aa effect on cell apoptosis by TUNEL staining. All data are means ± SEM, tested using one-way ANOVA with Tukey’s post hoc analysis. Fig. S6. Rsrc1-161aa affects the levels of reactive oxygen species. a. Representative images showing DHE staining of wild-type, Rsrc1+/+/circRsrc1−/− and rescue cells. Bar, 20 μm. b. ROS levels were quantified by measuring the average gray value of the DHE signal in cells by the ImageJ software. All data are means ± SEM, tested using one-way ANOVA with Tukey’s post hoc analysis; **P < 0.01. c. Western blotting showing protein levels in wild-type, Rsrc1+/+/circRsrc1−/− and rescue cells. Fig. S7. Rsrc1-161aa does not affect C1qbp interaction with the mitochondrial ribosome. a. RT-qPCR quantification of mtDNA amounts based on the ratio of mtDNA/nuclearDNA. All data are means ± SEM, tested using one-way ANOVA with Tukey’s post hoc analysis. b. RT-qPCR detection of certain gene transcripts of the mitochondrial respiratory chain in wild-type and Rsrc1+/+/circRsrc1−/− cells. RNA data were normalized to 18S levels. All data are means ± SEM, tested using a two-tailed unpaired t-test. c. circRsrc1 regulating the association of C1qbp to mitoribosomal proteins remained unchanged in Rsrc1+/+/circRsrc1−/− cells. d. Western blotting analysis of mitochondrial ribosomal proteins in wild-type, Rsrc1+/+/circRsrc1−/− and rescue cells. β-tubulin was the loading control. e. Schematic model. The loss of Rsrc1-161aa results in weakened binding ability between C1qbp and mitochondrial mRNA, as well as affecting the assembly of mitochondrial ribosomes, leading to decreased translation of mitochondrial proteins and ultimately causing damage to oxidative phosphorylation.

**Additional file 2: Table S1.** Results of analysis of circRNA sequencing.

**Additional file 3: Table S2.** Isotope-labeled heavy synthetic peptide sequence used for relative targeted quantification by PRM. **Table S3.** Primers used in PCR, RT-PCR and RT-qPCR. **Table S4.** Antibodies and dilution concentrations.

**Additional file 4.** Images of the original blots presented in this study.

**Additional file 5.** Individual data values.

## Data Availability

All data generated or analyzed during this study are included in this published article, its supplementary information files, and publicly available repositories. The uncropped gels/blots are provided in Additional file 4. Raw data for this study are provided in Additional file 5. The RNA-seq data were deposited in SRA (https://www.ncbi.nlm.nih.gov/sra/) under accession number SRR24075466. CIRI2 is freely available together with full documentation at https://sourceforge.net/projects/ciri/. The CIRCexplorer2 can be downloaded from https://github.com/YangLab/CIRCexplorer2. The find_circ can be downloaded from https://github.com/marvin-jens/find_circ. The mass spectrometry proteomics data have been deposited to the ProteomeXchange Consortium (http://proteomecentral.proteomexchange.org) via the iProX partner repository [54] with the dataset identifier PXD041424.

## Abbreviations

*circRNA*: Circular RNA
*Sry*: Sex-determining region Y
*Bp*: Base pairs
*Rsrc1*: Arginine/serine-rich coiled-coil 1
*C1qbp*: Complement component 1, q subcomponent binding protein
*GFP*: Green fluorescent protein
*GAPDH*: Glyceraldehyde-3-phosphate dehydrogenase
*Cdk1*: Cyclin-dependent kinase 1
*MtNd1*: NADH dehydrogenase 1, mitochondrial
*MtNd3*: NADH dehydrogenase 3, mitochondrial
*MtNd4*: NADH dehydrogenase 4, mitochondrial
*MtNd5*: NADH dehydrogenase 5, mitochondrial
*MtCytb*: Cytochrome b, mitochondrial
*MtCox1*: Cytochrome c oxidase subunit I
*MtCox2*: Cytochrome c oxidase subunit II
*Ndufa9*: NADH: ubiquinone oxidoreductase subunit A9
*Sdha*: Succinate dehydrogenase complex, subunit A, flavoprotein (Fp)
*Uqcrfs1*: Ubiquinol-cytochrome c reductase, Rieske iron-sulfur polypeptide 1
*Atpb*: Beta subunit of ATP synthase
*Vdac*: Voltage-dependent anion channel
*Mrps22*: Mitochondrial ribosomal protein S22
*Mrps29*: Mitochondrial ribosomal protein S29
*Mrpl3*: Mitochondrial ribosomal protein L3
*Mrpl28*: Mitochondrial ribosomal protein L28
*OXPHOS*: Oxidative phosphorylation
*OCR*: Oxygen consumption rate
*ECAR*: Extracellular acidification rate
*LDHC*: Lactate dehydrogenase C
*PDH*: Pyruvate dehydrogenase
