# Supplementary material for: A novel protein encoded by circRsrc1 regulates mitochondrial ribosome assembly and translation during spermatogenesis
Source: BMC Biol. 2023 Apr 24;21:94. doi: 10.1186/s12915-023-01597-z (PMC10127071; doi:10.1186/s12915-023-01597-z)
Supplement: Supplementary file 1 — Additional file 1: Fig. S1. Peptides translated by detected circRNAs are present in mouse testes. a. The specific peptides translated by circRNAs were detected by mass spectrometry and the corresponding spectra are shown. Fig. S2. The detected circRNAs are present in mouse testes. a. Linear splicing forms of the gene of interest were detected specifically in mouse testes by convergent primers. Both divergent primers detected circular forms in cDNA but not in gDNA of mouse testes. Sanger sequencing confirmed the presence of circular RNAs. Fig. S3. Different knockout mice were constructed using the CRISPR/Cas9 system. a. Relative expression levels of Rsrc1 in mouse testes at different ages in weeks. b. Relative expression levels of Rsrc1 in different germ cells and somatic cells of mouse testes. c. Subcellular localization of circRsrc1 in mouse testes was determined by RT-qPCR. 18S and U6 were used as internal controls. d. Schematic of the targeted exon 4 of mouse and sequence from wild-type and Rsrc1−/−/circRsrc1+/+ mouse. Sanger sequencing after PCR confirmed that the knockout was successful. e. Identification of Rsrc1−/−/circRsrc1+/+ mice by PCR. f. RT- PCR analysis of circRsrc1 and Rsrc1 mRNA levels in wild-type and Rsrc1−/−/circRsrc1+/+ mice. g. Western blotting of Rsrc1 protein in wild-type and Rsrc1−/−/circRsrc1+/+ mice. h. Schematic of the targeted intron of mouse and sequence from wild-type and Rsrc1+/+/circRsrc1−/− mouse. Sanger sequencing after PCR confirmed that the knockout was successful. i. Identification of Rsrc1+/+/circRsrc1−/− mice by PCR. j. RT-PCR analysis of circRsrc1 mRNA levels in wild-type and Rsrc1+/+/circRsrc1−/− mice. Sanger sequencing was performed after PCR. k. Schematic of the targeted intron and exon 3 of sequences from wild-type and Rsrc1−/−/circRsrc1−/− mouse. Sanger sequencing after PCR confirmed that the knockout was successful. l. RT-PCR analysis of circRsrc1 and Rsrc1 mRNA levels in wild-type and Rsrc1−/−/circRsrc1−/− mice. m. RT-q [file 12915_2023_1597_MOESM1_ESM.pdf]

Fig. S1

a

circAp2b1 VELSLPR

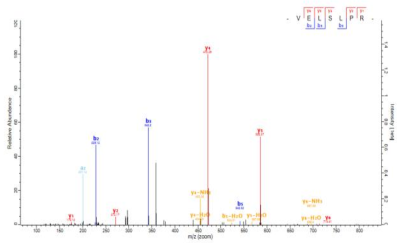

circNbas ASETEALR

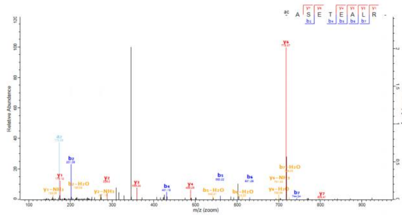

circClptm1 TSQALEGPHVSPAETYSPK

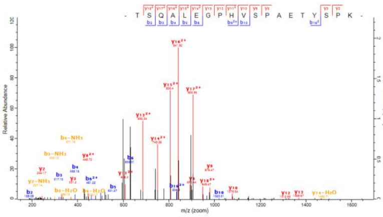

circRsph1 MGPGTPETMSK

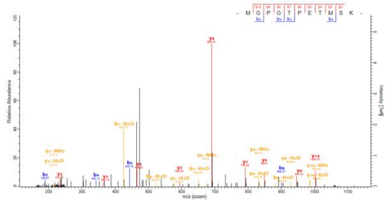

circLekr1 MKEIDMLNK

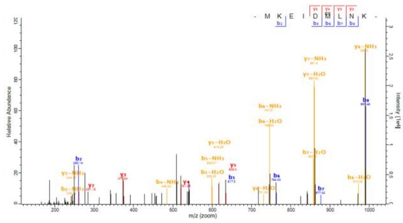

circSmarcc2 TANQQVSVQPGGAVAPVSGR

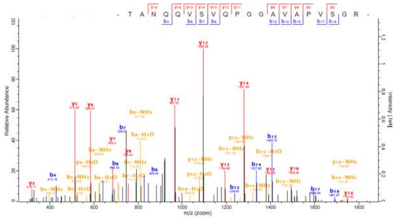

circLin9 MFHSSKISPMK

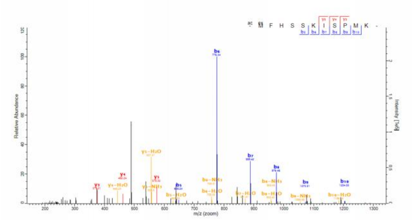

circZnrf2 NGLKSIDLVQSTPQTKHSR

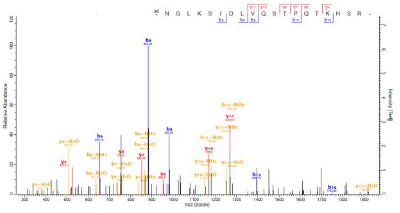

Fig. S2

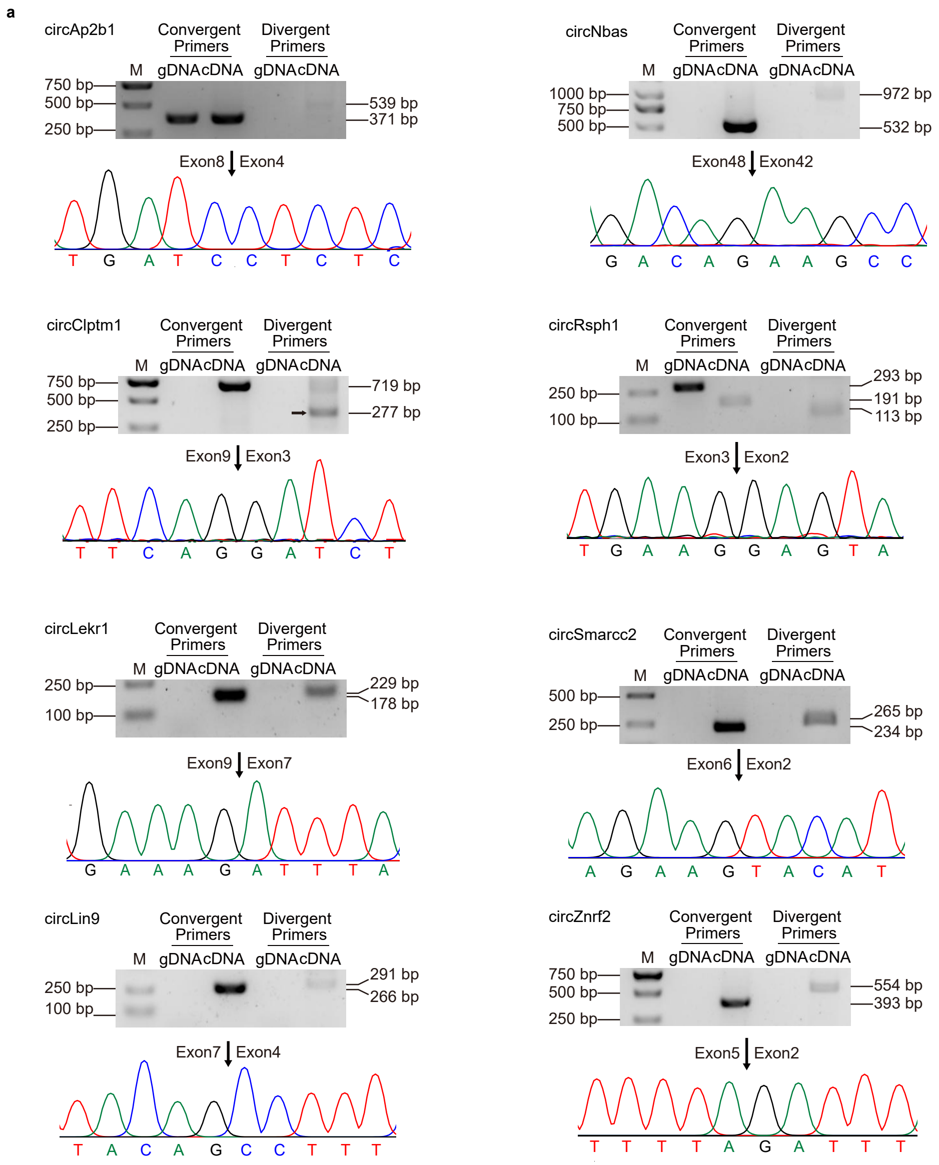
a
circAp2b1
Convergent Primers
Divergent Primers
M gDNA cDNA gDNA cDNA
750 bp
500 bp
250 bp
539 bp
371 bp
Exon8 Exon4
T G A T C C T C T C
circNbas
1000 bp
750 bp
500 bp
972 bp
532 bp
Exon48 Exon42
G A C A G A A G C C
circClptm1
750 bp
500 bp
250 bp
719 bp
277 bp
Exon9 Exon3
T T C A G G A T C T
circRsph1
250 bp
100 bp
293 bp
191 bp
113 bp
Exon3 Exon2
T G A A G G A G T A
circLekr1
250 bp
100 bp
229 bp
178 bp
Exon9 Exon7
G A A A G A T T T A
circSmarcc2
500 bp
250 bp
265 bp
234 bp
Exon6 Exon2
A G A A G T A C A T
circLin9
250 bp
100 bp
291 bp
266 bp
Exon7 Exon4
T A C A G C C T T T
circZnrf2
750 bp
500 bp
250 bp
554 bp
393 bp
Exon5 Exon2
T T T T A G A T T T

Fig. S3

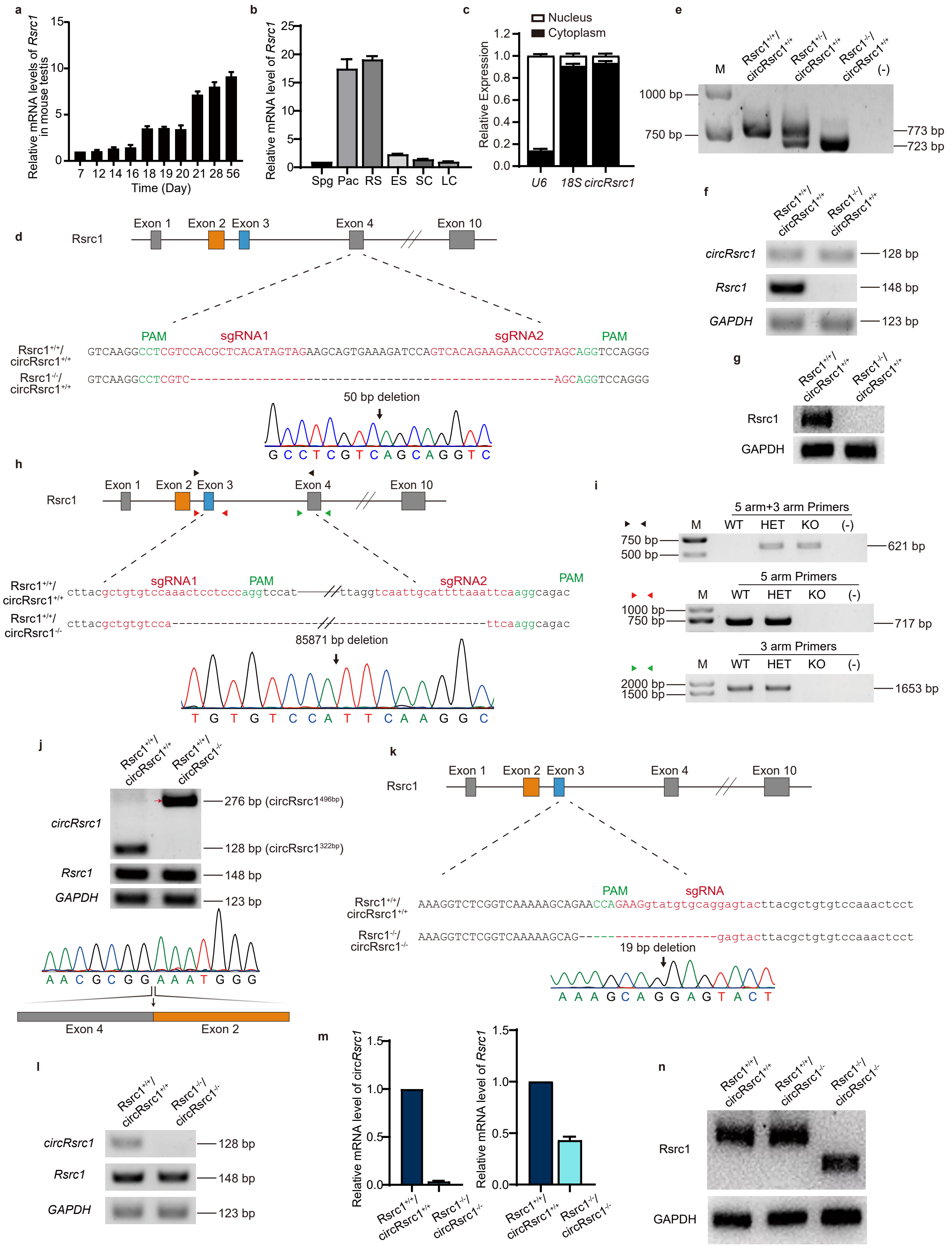

a
Relative mRNA levels of Rsrc1 in mouse testis
Time (Day)
7 12 14 16 18 19 20 21 28 56
b
Relative mRNA level of Rsrc1
Spg Pac RS ES SC LC
c
Nucleus
Cytoplasm
Relative Expression
U6 18S circRsrc1
d
Rsrc1
Exon 1 Exon 2 Exon 3 Exon 4 Exon 10
PAM sgRNA1 sgRNA2 PAM
Rsrc1+/+/ circRsrc1+/+
GTCAAGGCCTCGTCCACGCTCACATAGTAGAAGCAGTGAAAGATCCAGTCACAGAAGAACCCGTAGCAGGTCCAGGG
Rsrc1-/-/ circRsrc1+/+
GTCAAGGCCTCGTC----AGCAGGTCCAGGG
50 bp deletion
G C C T C G T C A G C A G G T C
e
M
Rsrc1+/+/ circRsrc1+/+
Rsrc1+/-/ circRsrc1+/+
Rsrc1-/-/ circRsrc1+/+
(-)
1000 bp
750 bp
773 bp
723 bp
f
circRsrc1
Rsrc1
GAPDH
128 bp
148 bp
123 bp
g
Rsrc1
GAPDH
h
Rsrc1
Exon 1 Exon 2 Exon 3 Exon 4 Exon 10
sgRNA1 PAM sgRNA2 PAM
Rsrc1+/+/ circRsrc1+/+
cttacgctgtgtccaaactcctcccaggtccat ttaggtcaattgcattttaaattcaaggcagac
Rsrc1+/+/ circRsrc1-/-
cttacgctgtgtcca----ttcaaggcagac
85871 bp deletion
T G T G T C C A T T C A A G G C
i
5 arm+3 arm Primers
M WT HET KO (-)
750 bp
500 bp
621 bp
5 arm Primers
1000 bp
750 bp
717 bp
3 arm Primers
2000 bp
1500 bp
1653 bp
j
Rsrc1+/+/ circRsrc1+/+
Rsrc1+/+/ circRsrc1-/-
circRsrc1
Rsrc1
GAPDH
276 bp (circRsrc1496bp)
128 bp (circRsrc1322bp)
148 bp
123 bp
A A C G C G G A A A T G G G
Exon 4
Exon 2
k
Rsrc1
Exon 1 Exon 2 Exon 3 Exon 4 Exon 10
PAM sgRNA
Rsrc1+/+/ circRsrc1+/+
AAAGGTCTCGGTCAAAAAGCAGAACCAGAAGgtatgtgcaggagtacttacgctgtgtccaaactcct
Rsrc1-/-/ circRsrc1-/-
AAAGGTCTCGGTCAAAAAGCAG----gagtacttacgctgtgtccaaactcct
19 bp deletion
A A A G C A G G A G T A C T
l
Rsrc1+/+/ circRsrc1+/+
Rsrc1-/-/ circRsrc1-/-
circRsrc1
Rsrc1
GAPDH
128 bp
148 bp
123 bp
m
Relative mRNA level of circRsrc1
Relative mRNA level of Rsrc1
Rsrc1+/+/ circRsrc1+/+
Rsrc1-/-/ circRsrc1-/-
n
Rsrc1+/+/ circRsrc1+/+
Rsrc1+/+/ circRsrc1-/-
Rsrc1-/-/ circRsrc1-/-
Rsrc1
GAPDH

Fig. S4

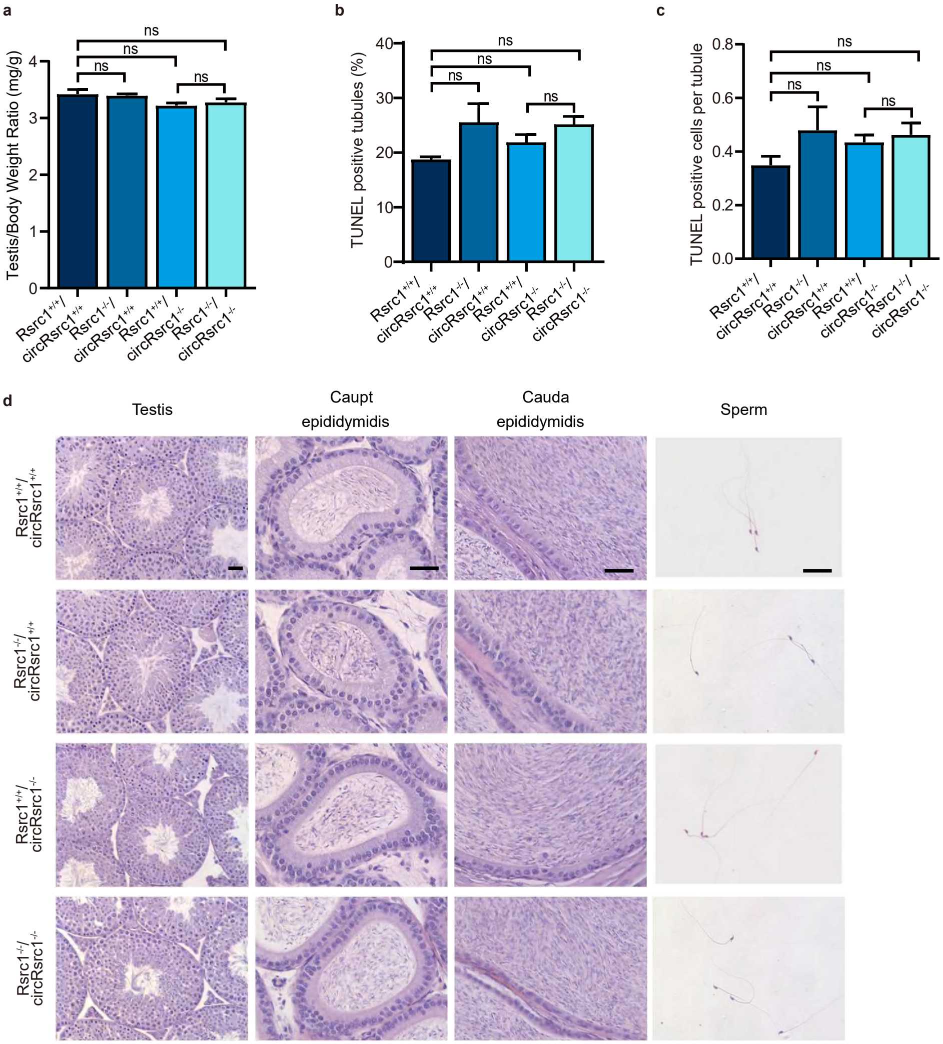
a
ns
ns
ns
ns
Testis/Body Weight Ratio (mg/g)
0
1
2
3
4
Rsrc1+/+/ circRsrc1+/+
Rsrc1-/-/ circRsrc1+/+
Rsrc1+/+/ circRsrc1-/-
Rsrc1-/-/ circRsrc1-/-
b
TUNEL positive tubules (%)
0
10
20
30
40
c
TUNEL positive cells per tubule
0.0
0.2
0.4
0.6
0.8
d
Testis
Caupt epididymidis
Cauda epididymidis
Sperm

Fig. S5

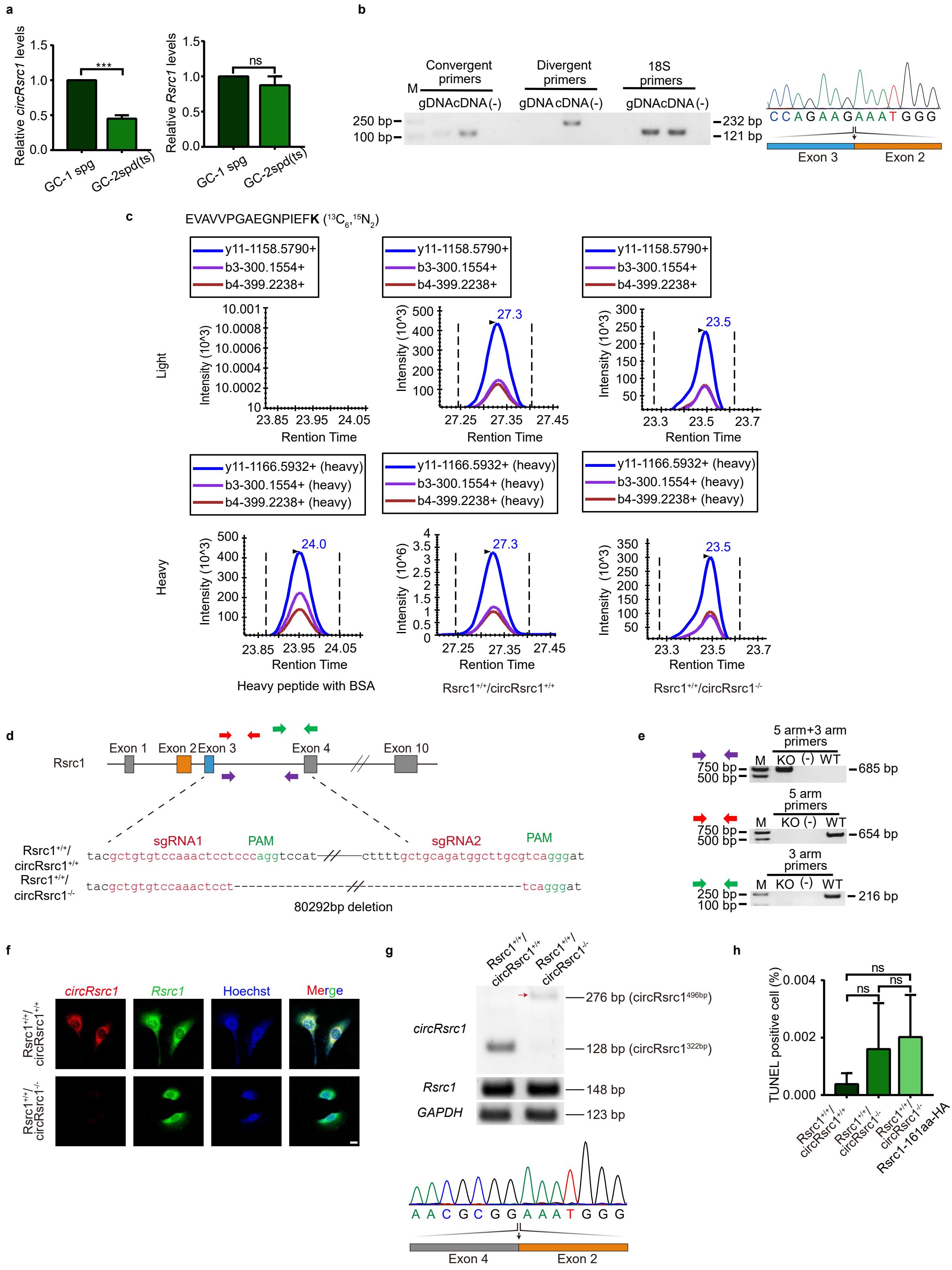

a
Relative circRsrc1 levels
1.5
1.0
0.5
0.0
***
GC-1 spg
GC-2spd(ts)
Relative Rsrc1 levels
ns
b
Convergent primers
Divergent primers
18S primers
M
gDNA cDNA (-)
250 bp
100 bp
232 bp
121 bp
C C A G A A G A A A T G G G
Exon 3
Exon 2
c
EVAVVPGAEGNPIEFK ($^{13}C_6$,$^{15}N_2$)
y11-1158.5790+
b3-300.1554+
b4-399.2238+
Light
Intensity (10^3)
Rention Time
27.3
23.5
y11-1166.5932+ (heavy)
b3-300.1554+ (heavy)
b4-399.2238+ (heavy)
Heavy
24.0
Intensity (10^6)
Heavy peptide with BSA
Rsrc1+/+/circRsrc1+/+
Rsrc1+/+/circRsrc1-/-
d
Rsrc1
Exon 1
Exon 2
Exon 3
Exon 4
Exon 10
sgRNA1
PAM
sgRNA2
Rsrc1+/+/ circRsrc1+/+
tacgctgtgtccaaactcctcccaggtccat—//—cttttgctgcagatggcttgcgtcagggat
Rsrc1+/+/ circRsrc1-/-
tacgctgtgtccaaactcct---------------//----------------------tcagggat
80292bp deletion
e
5 arm+3 arm primers
M KO (-) WT
750 bp
500 bp
685 bp
5 arm primers
654 bp
3 arm primers
250 bp
100 bp
216 bp
f
circRsrc1
Rsrc1
Hoechst
Merge
Rsrc1+/+/ circRsrc1+/+
Rsrc1+/+/ circRsrc1-/-
g
Rsrc1+/+/ circRsrc1+/+
Rsrc1+/+/ circRsrc1-/-
circRsrc1
276 bp (circRsrc1 496bp)
128 bp (circRsrc1 322bp)
Rsrc1
148 bp
GAPDH
123 bp
A A C G C G G A A A T G G G
Exon 4
Exon 2
h
TUNEL positive cell (%)
0.004
0.002
0.000
ns
ns
ns
Rsrc1+/+/ circRsrc1+/+
Rsrc1+/+/ circRsrc1-/-
Rsrc1+/+/ circRsrc1-/- Rsrc1-161aa-HA

Fig. S6

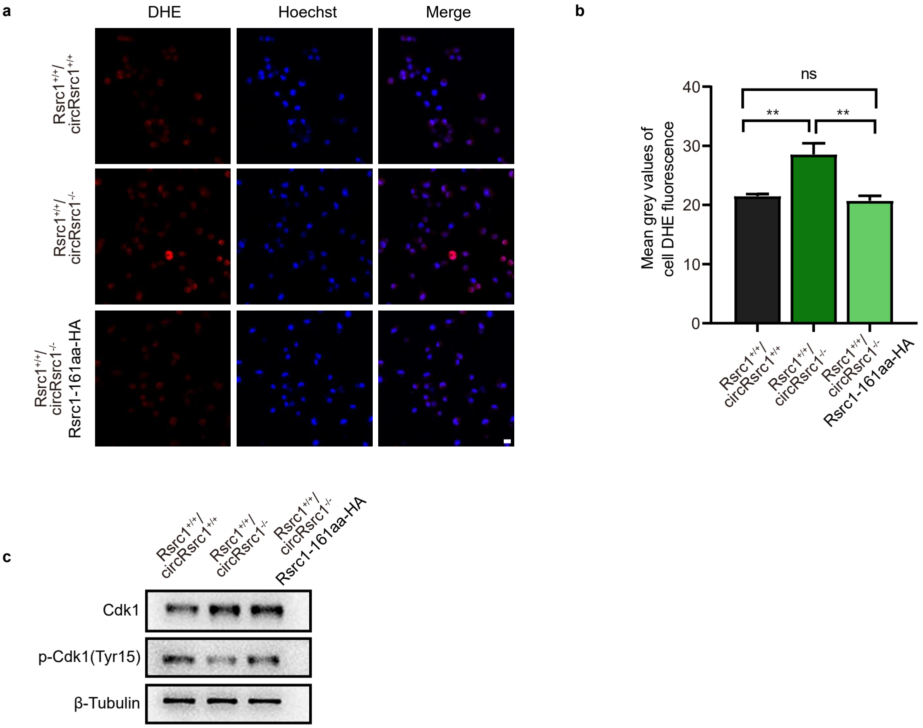
a
DHE
Hoechst
Merge
Rsrc1+/+/ circRsrc1+/+
Rsrc1+/+/ circRsrc1-/-
Rsrc1+/+/ circRsrc1-/- Rsrc1-161aa-HA
b
Mean grey values of cell DHE fluorescence
ns
**
**
0
10
20
30
40
Rsrc1+/+/ circRsrc1+/+
Rsrc1+/+/ circRsrc1-/-
Rsrc1+/+/ circRsrc1-/- Rsrc1-161aa-HA
c
Rsrc1+/+/ circRsrc1+/+
Rsrc1+/+/ circRsrc1-/-
Rsrc1+/+/ circRsrc1-/- Rsrc1-161aa-HA
Cdk1
p-Cdk1(Tyr15)
β-Tubulin

Fig. S7

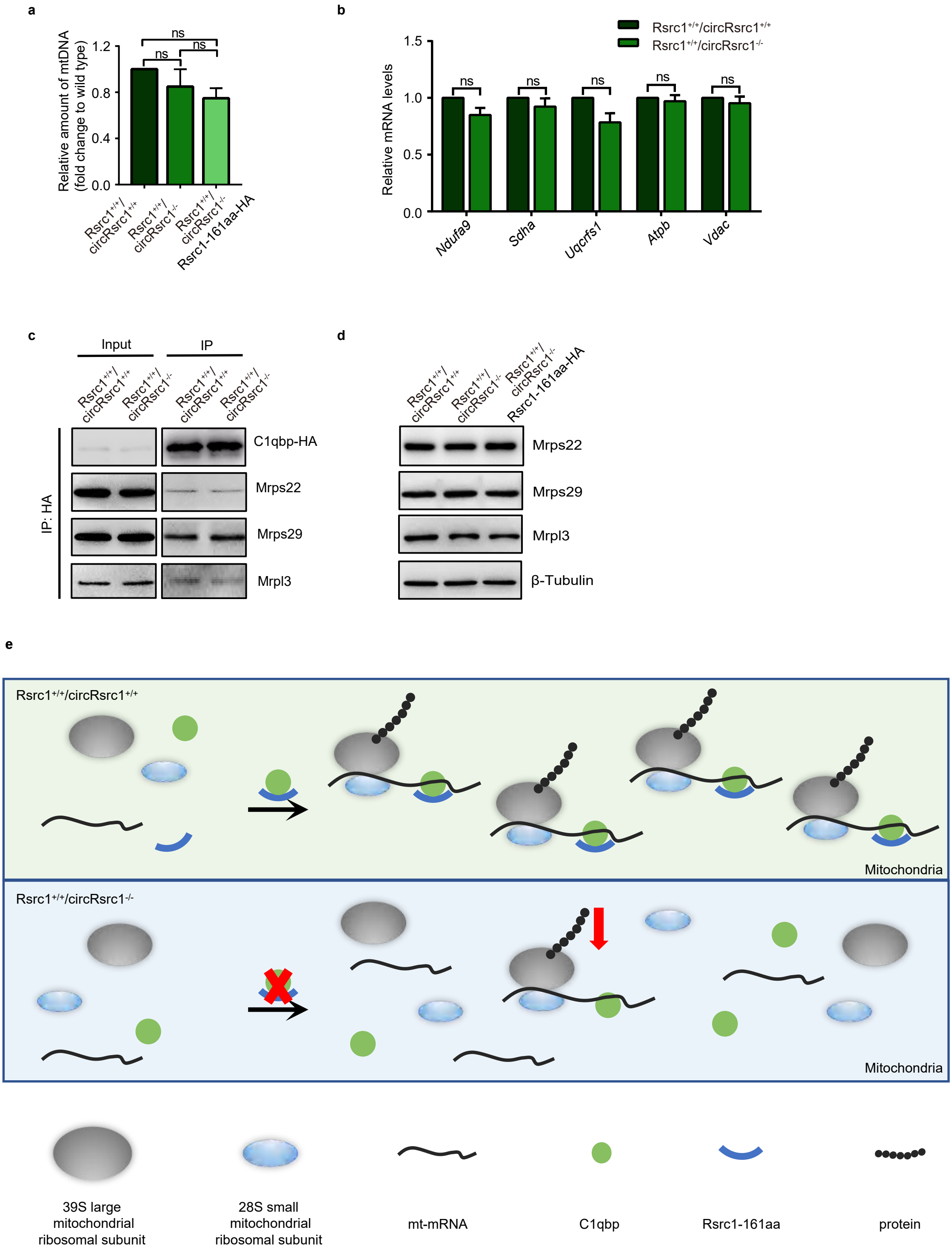
a
ns
ns
ns
1.2
0.8
0.4
0.0
Relative amount of mtDNA (fold change to wild type)
Rsrc1+/+/circRsrc1+/+
Rsrc1+/+/circRsrc1-/-
Rsrc1+/+/circRsrc1-/- Rsrc1-161aa-HA
b
Rsrc1+/+/circRsrc1+/+
Rsrc1+/+/circRsrc1-/-
1.5
1.0
0.5
0.0
Relative mRNA levels
Ndufa9
Sdha
Uqcrfs1
Atpb
Vdac
c
Input
IP
IP: HA
C1qbp-HA
Mrps22
Mrps29
Mrpl3
d
Rsrc1-161aa-HA
Mrps22
Mrps29
Mrpl3
β-Tubulin
e
Rsrc1+/+/circRsrc1+/+
Mitochondria
Rsrc1+/+/circRsrc1-/-
Mitochondria
39S large mitochondrial ribosomal subunit
28S small mitochondrial ribosomal subunit
mt-mRNA
C1qbp
Rsrc1-161aa
protein
