# Supplementary material for: A novel protein encoded by circRsrc1 regulates mitochondrial ribosome assembly and translation during spermatogenesis
Source: BMC Biol. 2023 Apr 24;21:94. doi: 10.1186/s12915-023-01597-z (PMC10127071; doi:10.1186/s12915-023-01597-z)
Supplement: Supplementary file 3 — Additional file 3: Table S2. Isotope-labeled heavy synthetic peptide sequence used for relative targeted quantification by PRM. Table S3. Primers used in PCR, RT-PCR and RT-qPCR. Table S4. Antibodies and dilution concentrations. [file 12915_2023_1597_MOESM3_ESM.pdf]

Table S2. Isotope-labeled heavy synthetic peptide sequence used for relative targeted quantification by PRM

Gene name	Peptide sequence	Precursor ion(m/z)	Purity grade
Rsrc1	VKEIEAIESDSFVQQTFR(13C6,15N4)	712.699749	Crude
Rsrc1	LQLVLEAAAK(13C6,15N2)	532.333698	Crude
Rsrc1	EIDPDSIPTAIK(13C6,15N2)	653.852649	Crude
CircRsrc1	EVAEEPGAEGNPIEFK(13C6,15N2)	555.297555	Crude
CircRsrc1	GLGQKAEPEEMGR(13C6,15N4)	706.347317	Crude
Mtnd1	GPNIVGPYGILQPFADAMK(13C6,15N2)	998.526235	Crude
Mtnd1	YSLFGALR(13C6,15N4)	468.762487	Crude
Mtnd2	LIYSTSLTMFPTNNNSK(13C6,15N2)	969.989683	Crude
Mtnd2	WIIITELMK(13C6,15N2)	577.840302	Crude
Mtnd3	ANPYECGFDPTSSAR(13C6,15N4)	841.361153	Crude
Mtnd3	ECGFDPTSSAR(13C6,15N4)LPF	797.365707	Crude
Mtnd4	ISIILDPLTK(13C6,15N2)	560.857006	Crude
Mtnd4	LTNHMINLQPSHTR(13C6,15N4)	557.958952	Crude
Mtnd5	SLNLSLK(13C6,15N2)	391.746728	Crude
Mtnd5	TDANTAALQAILYNR(13C6,15N4)	822.932603	Crude
Mtco1	AYFTSATMIIAIPTGVK(13C6,15N2)	896.493872	Crude
Mtco1	WLFSTNHK(13C6,15N2)	520.773808	Crude
Mtco2	VVLPMELPIR(13C6,15N4)	588.856061	Crude
Mtco2	LLEVDNR(13C6,15N4)	434.741751	Crude
Mtco3	EGTYQGHHTPIVQK(13C6,15N2)	534.941994	Crude
Mtatp6	LHSFQHWLVK(13C6,15N2)	651.863489	Crude
Mtatp6	QMMLIHTPK(13C6,15N2)	553.800654	Crude
Mt-Cyb	DVNYGWLIR(13C6,15N4)	573.302507	Crude
Mt-Cyb	KTHPLFK(13C6,15N2)	439.770537	Crude
Tubb2b	INVYYNEATGNK(13C6,15N2)	697.345523	Crude

Table S3. Primers used in PCR, RT-PCR and RT-qPCR

1. Primer sets used in RT-PCR analyses of mice testes

circRNA name	Primer name	Primer sequence(5'-3')	Expected product size (bp)
circRsrc1	circRsrc1-convergent-F	CTCTTCAGACAGCAGGACATACA	181
	circRsrc1-convergent-R	CCTGGAACGACTGCGACTTC	
	circRsrc1-divergent-F	CAGAACCAGAAGAAATGGGACGC	166
	circRsrc1-divergent-R	CAGGACCTTGAGTCGGACCT	
circAp2b1	circAp2b1-convergent-F	GCAGACTGACAACCTGGAACTAA	371
	circAp2b1-convergent-R	CTACAGCATTAGCCACCACCATT	
	circAp2b1-divergent-F	GAGCATCTGTGAGCGAGTAAC	539
	circAp2b1-divergent-R	GGTCTTCATCCTTCAAGCACTT	
circClptm1	circClptm1-convergent-F	ACGAGCACTTCACAGACTTCAA	719
	circClptm1-convergent-R	TTCCAGCAGAGCAACCTTCAC	
	circClptm1-divergent-F	GGCTCCATCTATATCCATGTCTA	277
	circClptm1-divergent-R	GCGTTGAAGTCTGTGAAGTG	
circLerk1	circLerk1-convergent-F	GTTGTCGGATGCAGCCATATTGAG	178
	circLerk1-convergent-R	CTCCTTACAGTTGTCCAGCTCCTT	
	circLerk1-divergent-F	ACCGTACAGGAAGAGCTGACAACA	229
	circLerk1-divergent-R	TGAAGTCTCAACCCAAGTGCCTCT	
circLin9	circLin9-convergent-F	CAGCAACGATGTCAACACCAGAT	266
	circLin9-convergent-R	TGCAGAAGAACATCTCCGAGGTT	
	circLin9-divergent-F	CCCGATGAAATCCCTCTACCC	291
	circLin9-divergent-R	AGTGGCTTGTCTATGTTTGAGTAG	
circNbas	circNbas-convergent-F	GCCACCTATCCAGATGCCTTG	532
	circNbas-convergent-R	GCTTGCCATCCTCCTCACTC	
	circNbas-divergent-F	GCCTCAGAGACAGAAGCCTTG	972
	circNbas-divergent-R	ACCGTGACAGATCGTAGAAGTAAC	
circRsph1	circRsph1-convergent-F	CGCAATGAGGTGGGAGAACGAC	191
	circRsph1-convergent-R	AAGGTGCCTTGGCCGTGCTT	
	circRsph1-divergent-F	CACGGCCAAGGCACCTTTATCTA	113
	circRsph1-divergent-R	CGTTGGGCAGTCGTGCTTTC	
circSmarcc2	circSmarcc2-convergent-F	GCCAACCAACAAGTCTCTGTCCAG	234
	circSmarcc2-convergent-R	ACATTGCGGTCCATGCGTGATG	
	circSmarcc2-divergent-F	GAACCTAGAAGAAGTACATACAAGCAGAAC	265
	circSmarcc2-divergent-R	ACATTGCGGTCCATGCGTGAT	
circZnrf2	circZnrf2-convergent-F	TACGGCTCACAGGACTCAGT	393
	circZnrf2-convergent-R	GTGCTCTGGACAAGATCTATTGAC	
	circZnrf2-divergent-F	CAATATGCCTTGAAGAACTG	554
	circZnrf2-divergent-R	TACTCAGCACATCCTCATTA	

2. Primer sets used in PCR analyses of mice genotypes

Mouse name	Primer name	Primer sequence(5'-3')	Expected product size (bp)
Rsrc1-/-/circRsrc1+/+	Rsrc1-/-/circRsrc1+/+-F	TTTCTGCTGGTTCTGTGGGATTCC	773
	Rsrc1-/-/circRsrc1+/+-R	AAACATGCAAGCCCTGACCTTCTT	
Rsrc1+/+/circRsrc1-/-	Rsrc1+/+/circRsrc1-/--5+3 arm-F	GTACAGCCTCAGTAATAGTGTCAG	621
	Rsrc1+/+/circRsrc1-/--5+3 arm-R	GTCTTTGCCTTTATCCTTCTCCTT	
	Rsrc1+/+/circRsrc1-/--5 arm-F	TGGTCCTCCTACAAGGTCACTCT	717

	Rsrc1+/+/circRsrc1-/--5 arm-R	AGATGAGCAGGGTCCAATGAGAAA	
	Rsrc1+/+/circRsrc1-/--3 arm-F	GGCTTCTACTTGCTTACCTGTGTT	1653
	Rsrc1+/+/circRsrc1-/--3 arm-R	TGTATCTCGCTTTGCCACTGTTAC	
Rsrc1-/-/circRsrc1-/-	Rsrc1-/-/circRsrc1-/--F	TGGTCCTCCTACAAGGTCACTCT	717
	Rsrc1-/-/circRsrc1-/--R	AGATGAGCAGGGTCCAATGAGAAA	

3. Primer sets used in RT-PCR and RT-qPCR analyses of mice testes

Primer name	Primer sequence(5'-3')	Expected product size (bp)
circRsrc1-F1	AGAAGTCGCAGTCGTTCCAGG	128
circRsrc1-R1	TCCTCTTGCTTCTGCTTTCCTCTT	
circRsrc1-F2	CAGAACCAGAAGAAATGGGACGC	166
circRsrc1-R2	CAGGACCTTGAGTCGGACCT	
Rsrc1-F	GTAGCAGGTCCAGGGACAGAGA	148
Rsrc1-R	GGAGGCAGATGTTCTAATCCAGCT	
U6-F	ACCCTGAGAAATACCCTCACAT	62
U6-R	GACGACTGAGCCCCTGATG	
Cox2-F	ACGAAATCAACAACCCCGTA	172
Cox2-R	GGCAGAACGACTCGGTTATC	
GAPDH-F	AGGTCGGTGTGAACGGATTTG	123
GAPDH-R	TGTAGACCATGTAGTTGAGGTCA	
18S-F	TAACGAACGAGACTCTGGCAT	138
18S-R	CGGACATCTAAGGGCATCACAG	

4. Primer sets used in RT-qPCR analyses of cell samples

Primer name	Primer sequence(5'-3')
circRsrc1-divergent-F	AAGAAGTCGCAGTCGTTCCAG
circRsrc1-divergent-R	CAGGACCTTGAGTCGGACCT
circRsrc1-convergent-F	GAACCCGTAGCAGGTCCAG
circRsrc1-convergent-R	CCAGAATCCCCGCGTTTGAT
Gapdh-F	AGGTCGGTGTGAACGGATTTG
Gapdh-R	TGTAGACCATGTAGTTGAGGTCA
Ndufa9-F	GTCCGCTTTCGGGTTGTTAGA
Ndufa9-R	CCTCCTTTCCCGTGAGGTA
Sdha-F	GCGGTGGTCACCTTGATCC
Sdha-R	CCTCTGTAGAAGCGTCTGAATG
Uqcrfs1-F	GAGCCACCTGTTCTGGATGTG
Uqcrfs1-R	GCACGACGATAGTCAGAGAAGTC
Atpb-F	GGTTCATCCTGCCAGAGACTA
Atpb-R	AATCCCTCATCGAACTGGACG
Vdac-F	CCCACATACGCCGATCTTGG
Vdac-R	GTGGTTTCCGTGTTGGCAGA
Nd1-F	GGATCCGAGCATCTTATCCA
Nd1-R	GGTGGTACTCCCGCTGTAAA
Nd2-F	AGGGATCCCACTGCACATAG
Nd2-R	CTCCTCATGCCCCTATGAAA
Nd3-F	TTCGACCCTACAAGCTCTGC
Nd3-R	TGAATTGCTCATGGTAGTGGA

Nd4-F	CCACTGCTAATTGCCCTCAT
Nd4-R	CTTCAACATGGGCTTTTGGT
Nd5-F	TCCTACTGGTCCGATTCCAC
Nd5-R	TTTGATGTCGTTTTGGGTGA
Nd6-F	CGATCCACCAAACCCTAAAA
Nd6-R	TTGGTTGTCTTGGGTTAGCA
Cox1-F	GGTCAACCAGGTGCACTTTT
Cox1-R	TGGGGCTCCGATTATTAGTG
Cox2-F	ACGAAATCAACAACCCCGTA
Cox2-R	GGCAGAACGACTCGGTTATC
Cox3-F	CAAGGCCACCACACTCCTAT
Cox3-R	ATTCCTGTTGGAGGTCAGCA
Atp6-F	CCTTCCACAAGGAACTCCAA
Atp6-R	GGTAGCTGTTGGTGGGCTAA
Cytb-F	TGAGGGGGCTTCTCAGTAGA
Cytb-R	CTGTTTCGTGGAGGAAGAGG
18S-F	TAACGAACGAGACTCTGGCAT
18S-R	CGGACATCTAAGGGCATCACAG

5. Primer sets used in RT-PCR analyses of cell samples

Primer name	Primer sequence(5'-3')	Expected product size (bp)
circRsrc1-convergent-F	GAACCCGTAGCAGGTCCAG	121
circRsrc1-convergent-R	CCAGAATCCCCGCGTTTGAT	
circRsrc1-divergent-F	AAGAAGTCGCAGTCGTTCCAG	232
circRsrc1-divergent-R	CAGGACCTTGAGTCGGACCT	
18S-F	TAACGAACGAGACTCTGGCAT	138
18s-R	CGGACATCTAAGGGCATCACAG	
circRsrc1-F	AGAAGTCGCAGTCGTTCCAGG	128
circRsrc1-R	TCCTCTTGCTTCTGCTTTCCTCTT	
Rsrc1-F	GTAGCAGGTCCAGGGACAGAGA	148
Rsrc1-R	GGAGGCAGATGTTCTAATCCAGCT	
GAPDH-F	AGGTCGGTGTGAACGGATTTG	123
GAPDH-R	TGTAGACCATGTAGTTGAGGTCA	

6. Primer sets used in PCR analyses of cell genotypes

Primer name	Primer sequence(5'-3')	Expected product size (bp)
Rsrc1+/+/circRsrc1-/--5+3 arm-F	GTTGGTCCTCCTACAAGGTCACT	685
Rsrc1+/+/circRsrc1-/--5+3 arm-R	AGAGTCAGTAACACAGTGTGAGGT	
Rsrc1+/+/circRsrc1-/--5 arm-F	GTACAGCCTCAGTAATAGTGTCAG	654
Rsrc1+/+/circRsrc1-/--5 arm-R	CTTCCACCATCATCCAGAGAATG	
Rsrc1+/+/circRsrc1-/--3 arm-F	GGGTCATGTCATGTTTCAATCTCT	216
Rsrc1+/+/circRsrc1-/--3 arm-R	ACAAACAGAGTCAGTAACACAGTG	

Table S4. Antibodies and dilution concentrations

Antibody	Supplier	RRID	Cat#	Dilution (WB)
HA	MBL	AB_10951811	M180-3	1:1000 IF, IP :1:100
β-Tubulin	Abways	AB_10971396	AB0039	1:5000
Rsrc1	Invitrogen	AB_2720896	PA5-77169	1:1000
GAPDH	Abways	AB_10971398	AB0036	1:5000
C1QBP	Sino Biological	AB_2927404	50199-T62	1:1000 IF 1:500 IP 1:50
GFP	Abways	AB_2333135	AB0005	1:5000
GFP	EnoGene	AB_2927405	E12-026	IP, 1:50
Cdk1	Beyotime	AB_2927406	AF1516	1:1000
p-Cdk1(Tyr15)	Cell Signaling Technology	AB_2927409	4539T	1:1000
NDUFA9	Invitrogen	AB_1501787	459100	1:1000
SDHA	Invitrogen	AB_1607896	459200	1:5000
UQCRFS1	Invitrogen	AB_2725632	MA5-27471	1:1000
MTCO1	Invitrogen	AB_2532240	459600	1:1000
ATPB	Proteintech	AB_2061878	17247-1-AP	1:1000
VDAC	Cell Signaling Technology	AB_2927410	4866T	1:1000
MRPL3	Abcam	AB_2927411	ab151326	1:1000
MRPL28	Proteintech	AB_10859785	21604-1-AP	1:1000
MRPS22	Proteintech	AB_2146488	10984-1-AP	1:1000
MRPS29	BD Biosciences	AB_2927412	610662	1:1000
normal rabbit IgG	Beyotime	AB_2905533	A7016	IP, 1:50
normal rabbit IgG	Cell Signaling Technology	AB_1031062	2729S	IF, 1:100
