# Supplementary material for: A novel protein encoded by circRsrc1 regulates mitochondrial ribosome assembly and translation during spermatogenesis
Source: BMC Biol. 2023 Apr 24;21:94. doi: 10.1186/s12915-023-01597-z (PMC10127071; doi:10.1186/s12915-023-01597-z)
Supplement: Supplementary file 4 — Additional file 4. Images of the original blots presented in this study. [file 12915_2023_1597_MOESM4_ESM.pdf]

Fig. 1c

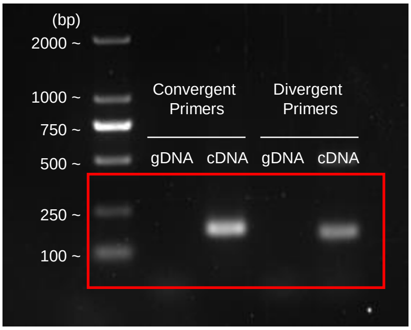
(bp)
2000 ~
1000 ~
750 ~
500 ~
250 ~
100 ~
Convergent Primers
Divergent Primers
gDNA
cDNA
gDNA
cDNA

Fig. 1f

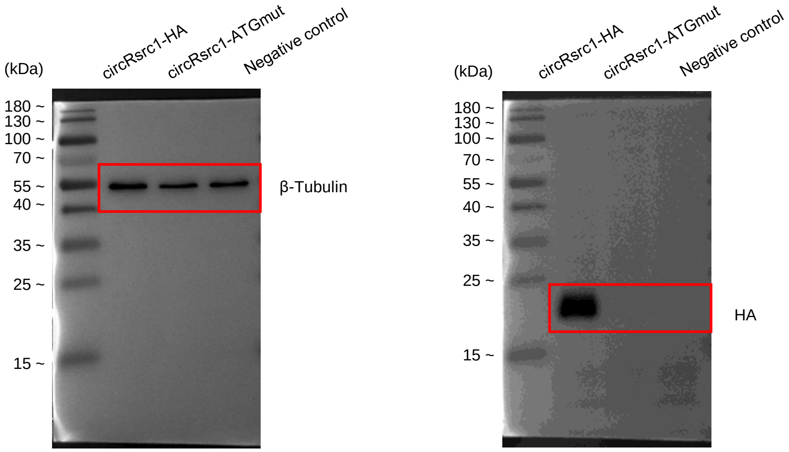
circRsrc1-HA
circRsrc1-ATGmut
Negative control
(kDa)
180 ~
130 ~
100 ~
70 ~
55 ~
40 ~
35 ~
25 ~
15 ~
β-Tubulin
circRsrc1-HA
circRsrc1-ATGmut
Negative control
(kDa)
180 ~
130 ~
100 ~
70 ~
55 ~
40 ~
35 ~
25 ~
15 ~
HA

Fig. 2a

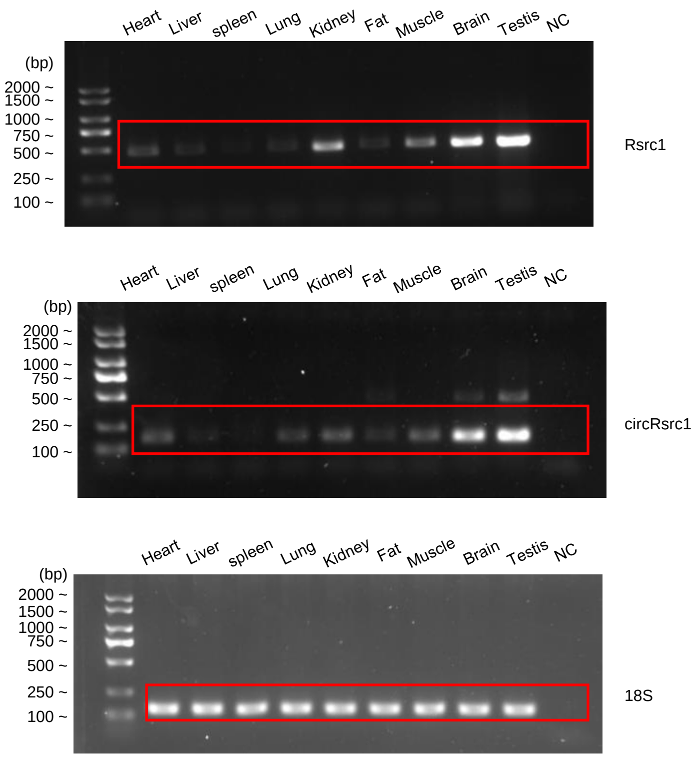

Heart
Liver
spleen
Lung
Kidney
Fat
Muscle
Brain
Testis
NC
(bp)
2000 ~
1500 ~
1000 ~
750 ~
500 ~
250 ~
100 ~
Rsrc1
circRsrc1
18S

Fig. 3f

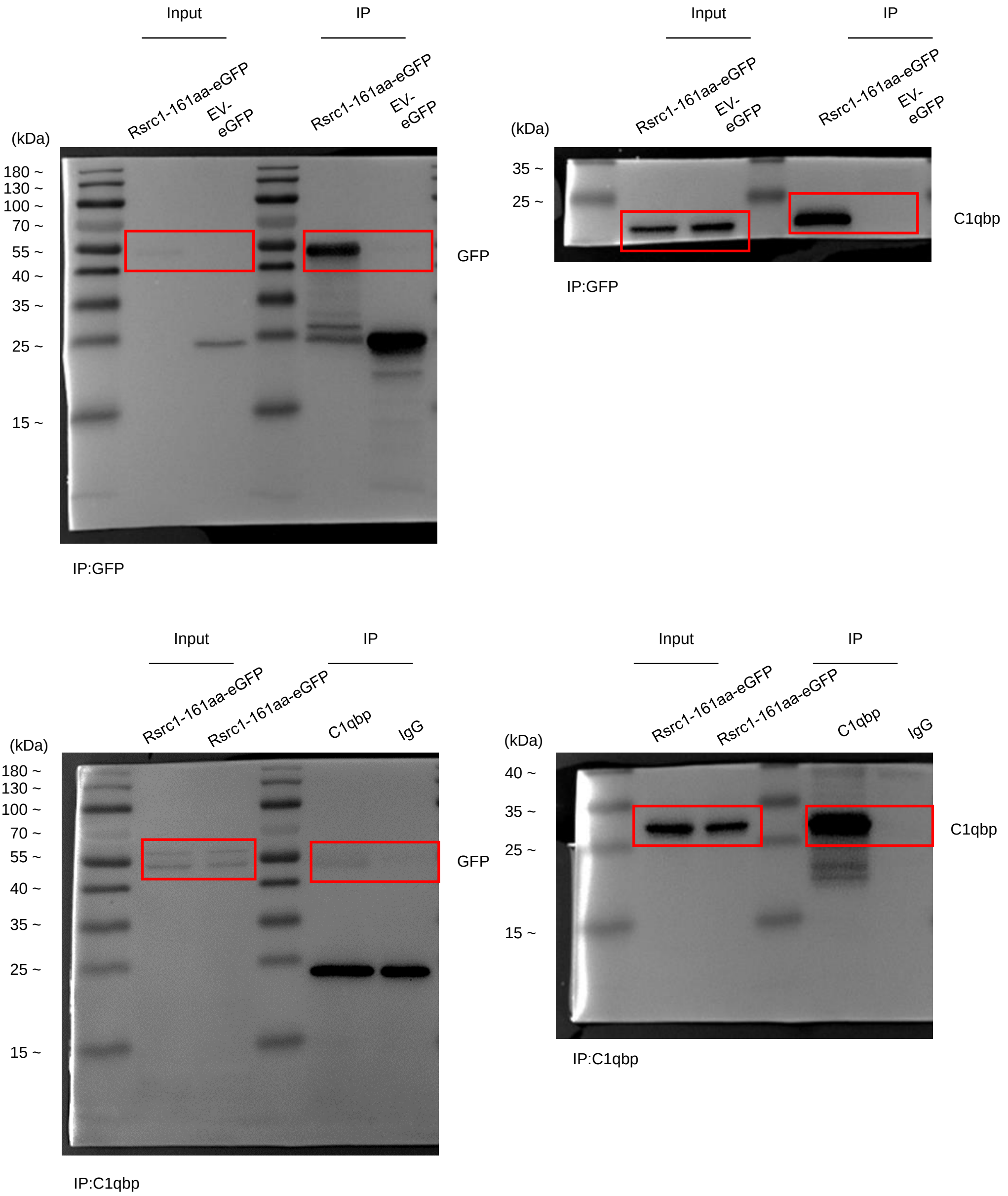

Input
IP
Rsrc1-161aa-eGFP
EV-eGFP
Rsrc1-161aa-eGFP
EV-eGFP
(kDa)
180 ~
130 ~
100 ~
70 ~
55 ~
40 ~
35 ~
25 ~
15 ~
GFP
IP:GFP
Input
IP
Rsrc1-161aa-eGFP
EV-eGFP
Rsrc1-161aa-eGFP
EV-eGFP
(kDa)
35 ~
25 ~
C1qbp
IP:GFP
Input
IP
Rsrc1-161aa-eGFP
Rsrc1-161aa-eGFP
C1qbp
IgG
(kDa)
180 ~
130 ~
100 ~
70 ~
55 ~
40 ~
35 ~
25 ~
15 ~
GFP
IP:C1qbp
Input
IP
Rsrc1-161aa-eGFP
Rsrc1-161aa-eGFP
C1qbp
IgG
(kDa)
40 ~
35 ~
25 ~
15 ~
C1qbp
IP:C1qbp

Fig. 5c

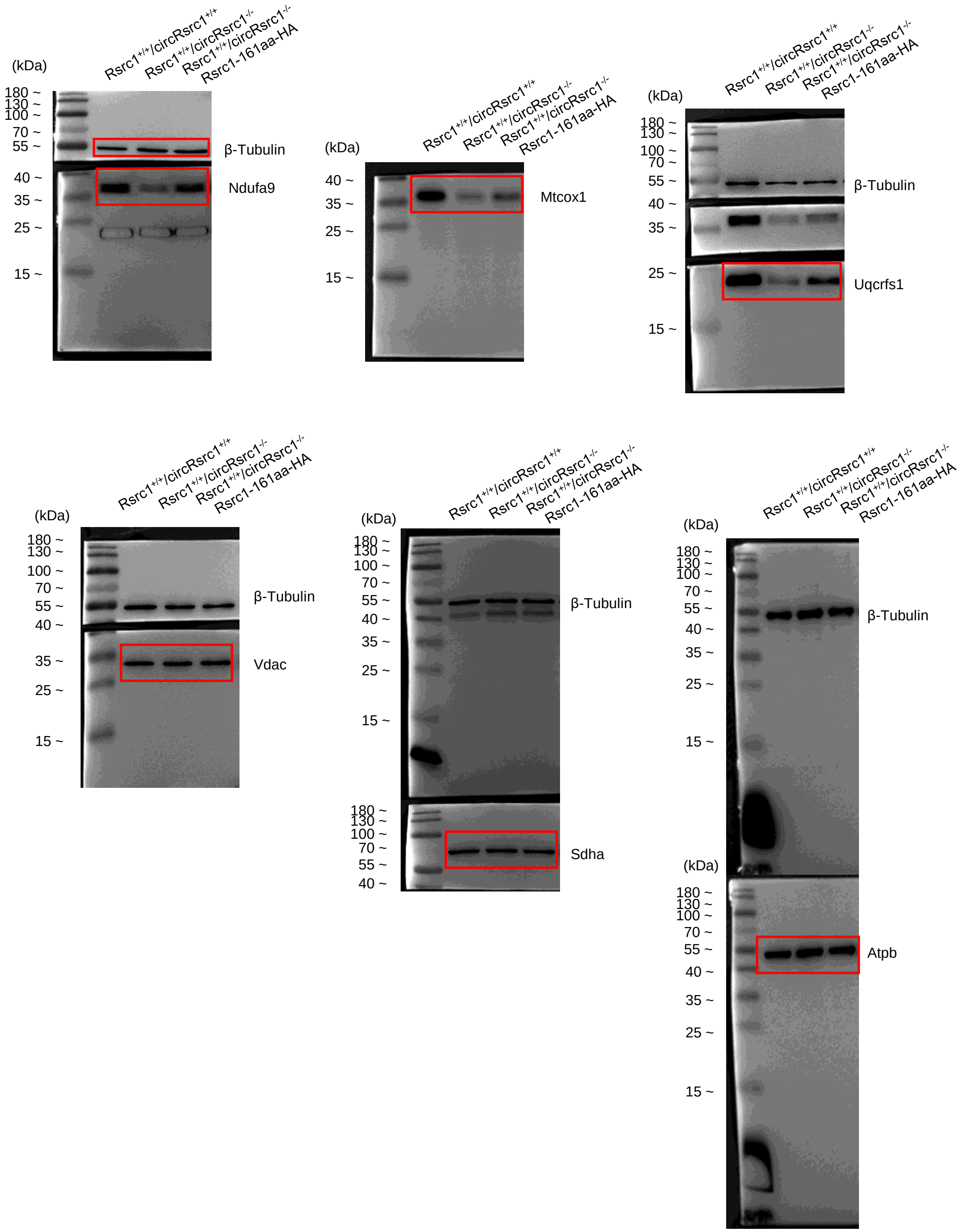

Rsrc1+/+/circRsrc1+/+
Rsrc1+/+/circRsrc1-/-
Rsrc1+/+/circRsrc1-/-
Rsrc1-161aa-HA
(kDa)
180 ~
130 ~
100 ~
70 ~
55 ~
40 ~
35 ~
25 ~
15 ~
β-Tubulin
Ndufa9
Mtcox1
Uqcrfs1
Vdac
Sdha
Atpb

Fig. 5d

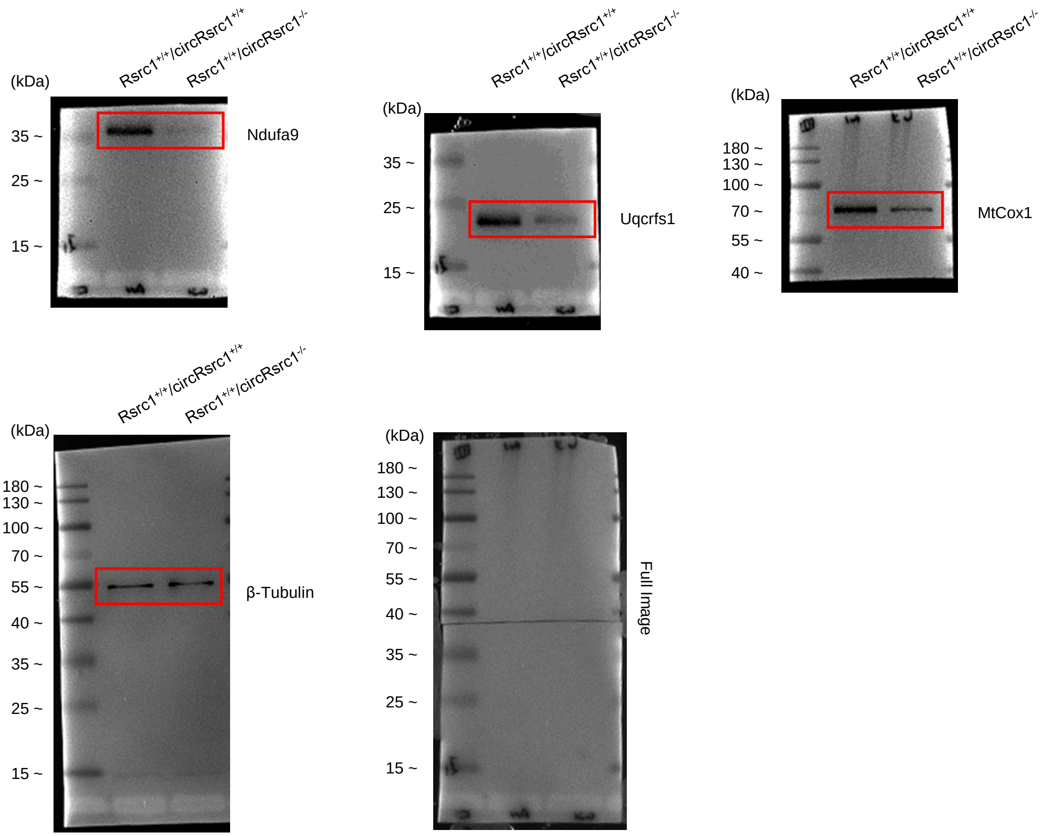

(kDa)
Rsrc1+/+/circRsrc1+/+
Rsrc1+/+/circRsrc1-/-
35 ~
25 ~
15 ~
Ndufa9
35 ~
25 ~
15 ~
Uqcrfs1
180 ~
130 ~
100 ~
70 ~
55 ~
40 ~
MtCox1
180 ~
130 ~
100 ~
70 ~
55 ~
40 ~
35 ~
25 ~
15 ~
β-Tubulin
Full Image

Fig. 5e

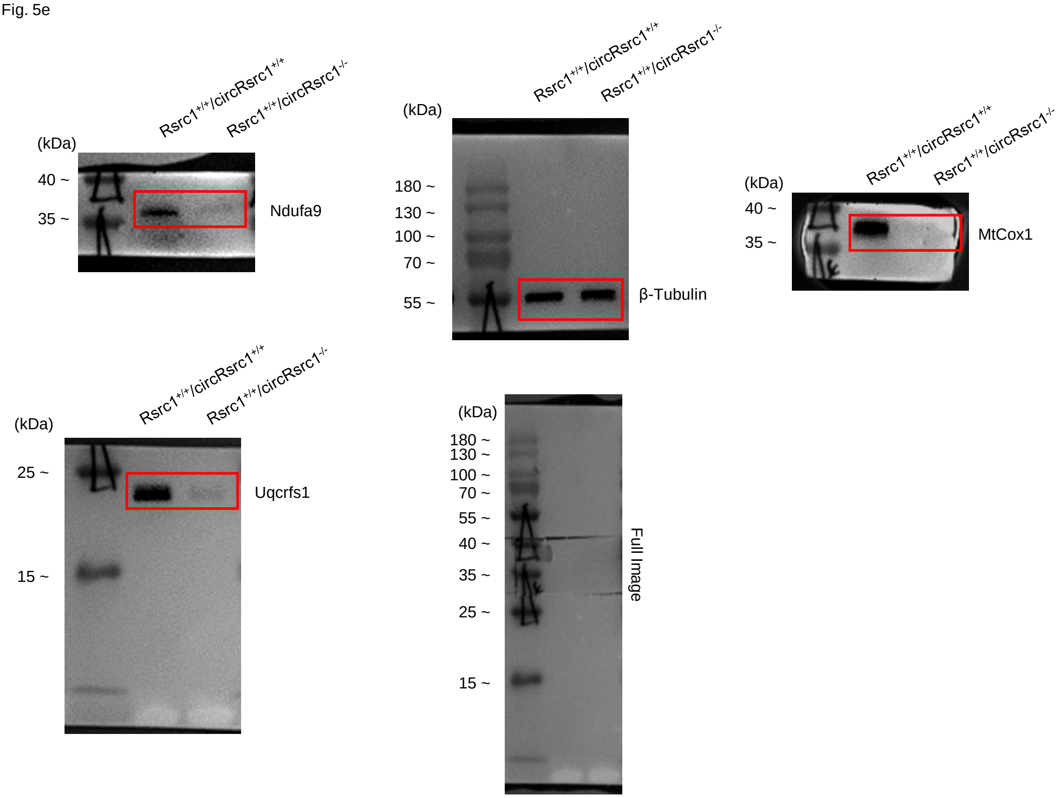

Rsrc1+/+/circRsrc1+/+
Rsrc1+/+/circRsrc1-/-
(kDa)
40 ~
35 ~
Ndufa9
(kDa)
180 ~
130 ~
100 ~
70 ~
55 ~
β-Tubulin
(kDa)
40 ~
35 ~
MtCox1
(kDa)
25 ~
15 ~
Uqcrfs1
(kDa)
180 ~
130 ~
100 ~
70 ~
55 ~
40 ~
35 ~
25 ~
15 ~
Full Image

Fig. 5f

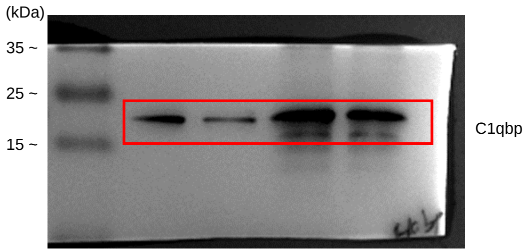

(kDa)
35 ~
25 ~
15 ~
C1qbp

Fig. 5g

Rsrc1$^{+/+}$/circRsrc1$^{+/+}$

(kDa)
180 ~
130 ~
100 ~
70 ~
55 ~
40 ~
35 ~
25 ~
15 ~

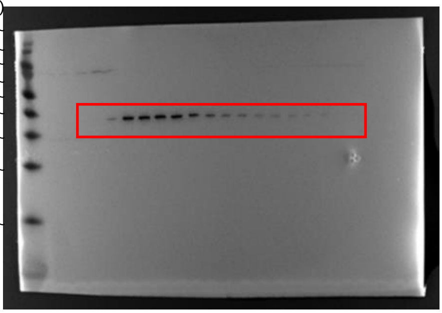

Mrps22

(kDa)
180 ~
130 ~
100 ~
70 ~
55 ~
40 ~
35 ~
25 ~
15 ~

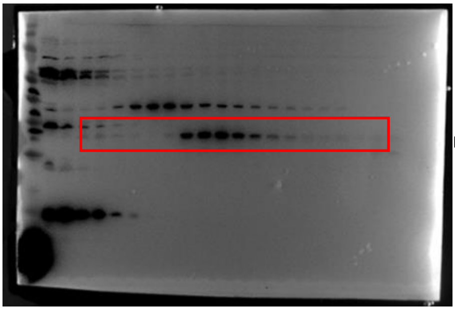

Mrpl28

Rsrc1$^{+/+}$/circRsrc1$^{-/-}$

(kDa)
180 ~
130 ~
100 ~
70 ~
55 ~
40 ~
35 ~
25 ~
15 ~

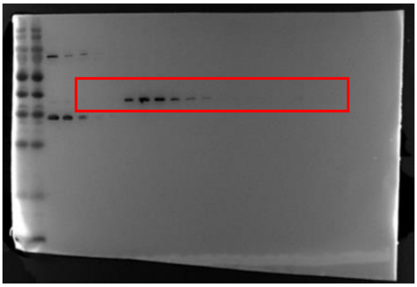

Mrps22

(kDa)
180 ~
130 ~
100 ~
70 ~
55 ~
40 ~
35 ~
25 ~
15 ~

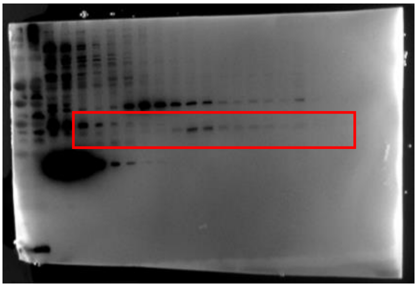

Mrpl28

Rsrc1$^{+/+}$/circRsrc1$^{-/-}$
Rsrc1-161aa-HA

(kDa)
180 ~
130 ~
100 ~
70 ~
55 ~
40 ~
35 ~
25 ~
15 ~

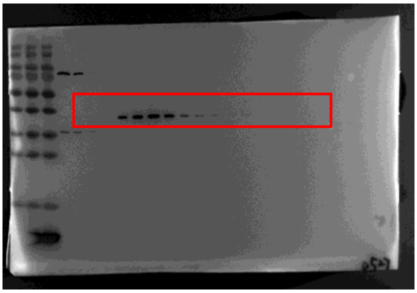

Mrps22

(kDa)
180 ~
130 ~
100 ~
70 ~
55 ~
40 ~
35 ~
25 ~
15 ~

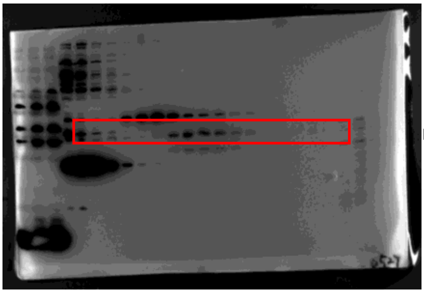

Mrpl28

Fig. S2

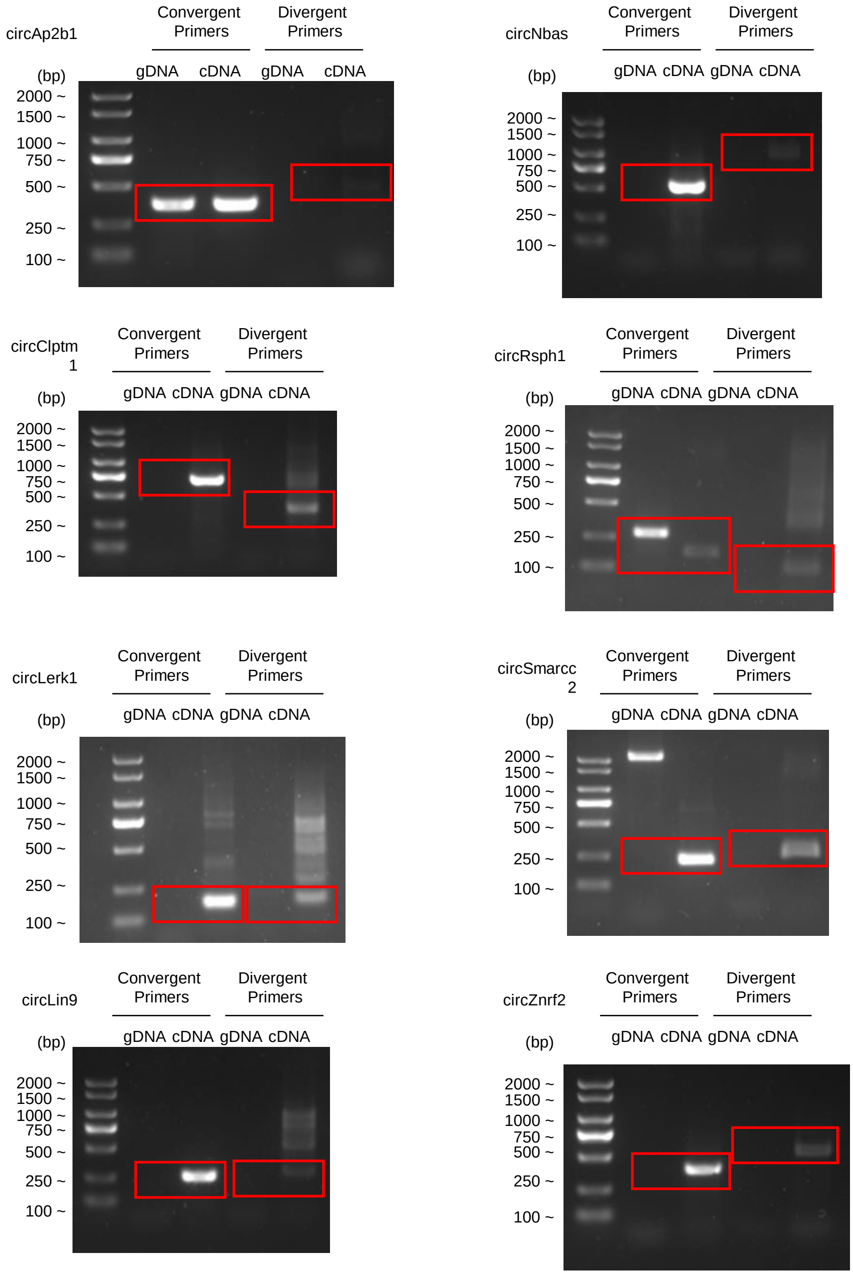
circAp2b1
Convergent Primers
Divergent Primers
(bp)
gDNA cDNA gDNA cDNA
2000 ~
1500 ~
1000 ~
750 ~
500 ~
250 ~
100 ~
circNbas
Convergent Primers
Divergent Primers
(bp)
gDNA cDNA gDNA cDNA
2000 ~
1500 ~
1000 ~
750 ~
500 ~
250 ~
100 ~
circClptm 1
Convergent Primers
Divergent Primers
(bp)
gDNA cDNA gDNA cDNA
2000 ~
1500 ~
1000 ~
750 ~
500 ~
250 ~
100 ~
circRsph1
Convergent Primers
Divergent Primers
(bp)
gDNA cDNA gDNA cDNA
2000 ~
1500 ~
1000 ~
750 ~
500 ~
250 ~
100 ~
circLerk1
Convergent Primers
Divergent Primers
(bp)
gDNA cDNA gDNA cDNA
2000 ~
1500 ~
1000 ~
750 ~
500 ~
250 ~
100 ~
circSmarcc 2
Convergent Primers
Divergent Primers
(bp)
gDNA cDNA gDNA cDNA
2000 ~
1500 ~
1000 ~
750 ~
500 ~
250 ~
100 ~
circLin9
Convergent Primers
Divergent Primers
(bp)
gDNA cDNA gDNA cDNA
2000 ~
1500 ~
1000 ~
750 ~
500 ~
250 ~
100 ~
circZnrf2
Convergent Primers
Divergent Primers
(bp)
gDNA cDNA gDNA cDNA
2000 ~
1500 ~
1000 ~
750 ~
500 ~
250 ~
100 ~

Fig. S3e

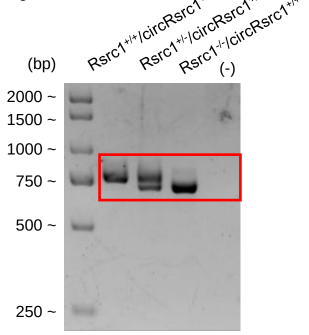

Fig. S3f

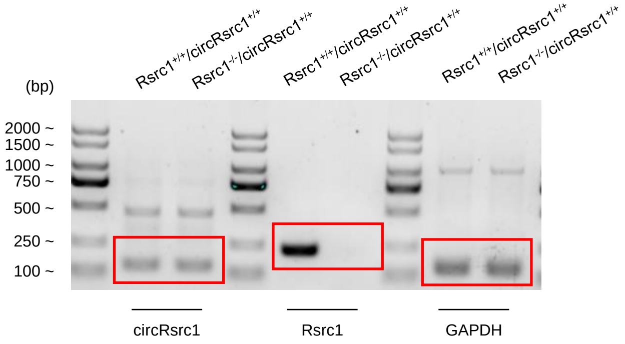

Fig. S3g

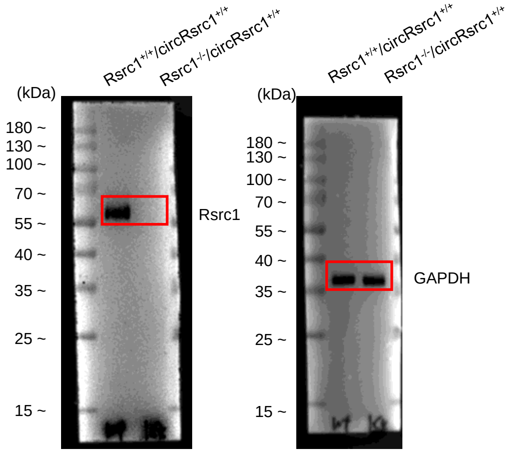

Fig. S3i

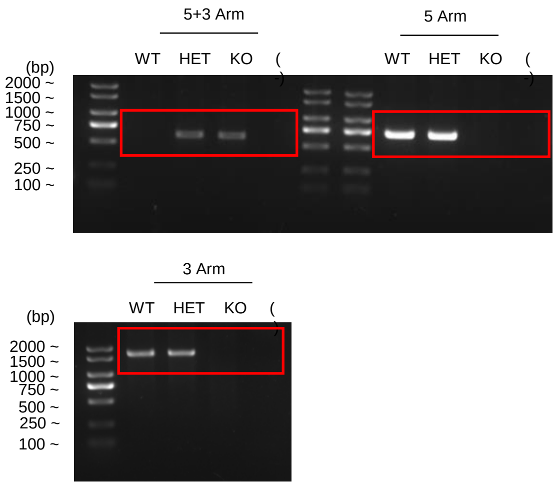

Fig. S3j

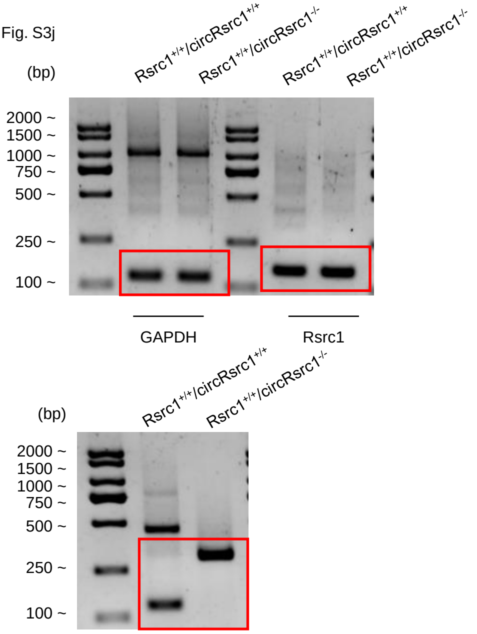

Fig. S3l

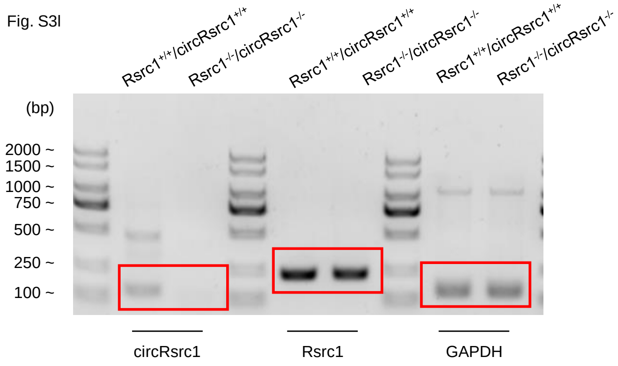

Fig. S3n

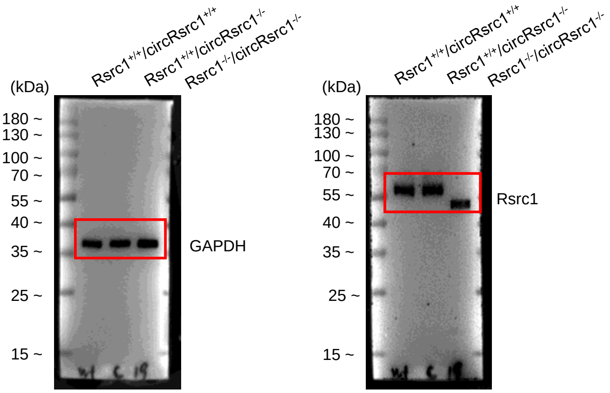

Fig. S5b

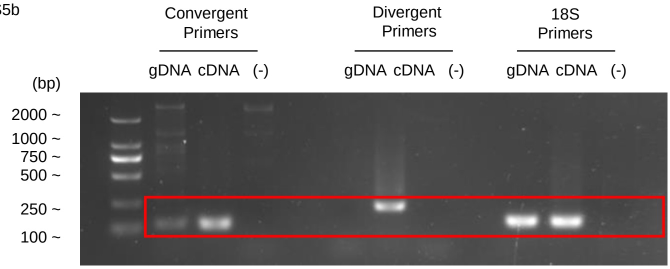

Fig. S5e

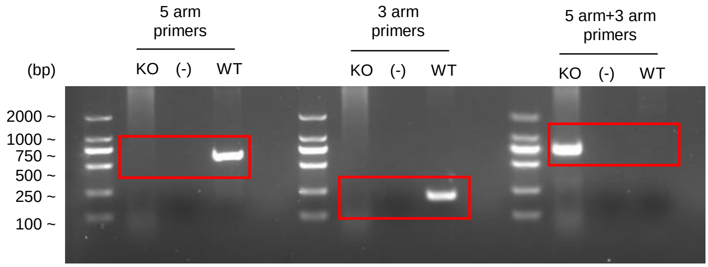

Fig. S5g

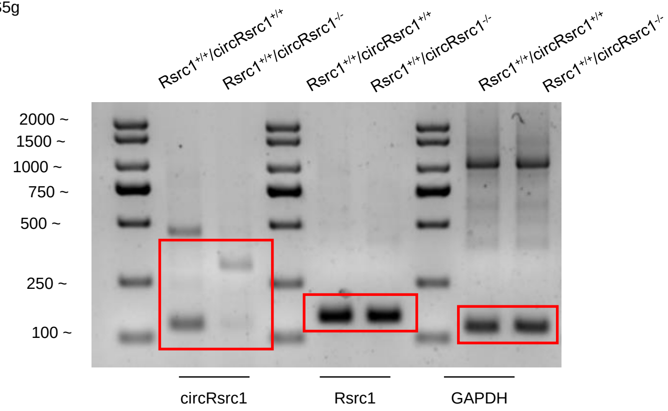

Fig. S6c

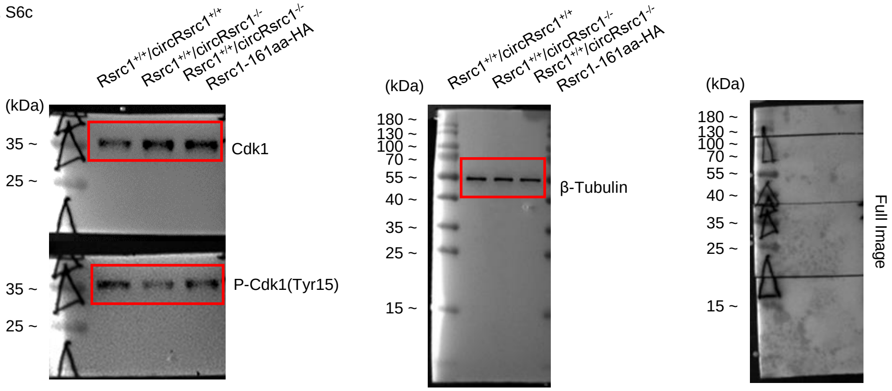

Fig. S7c

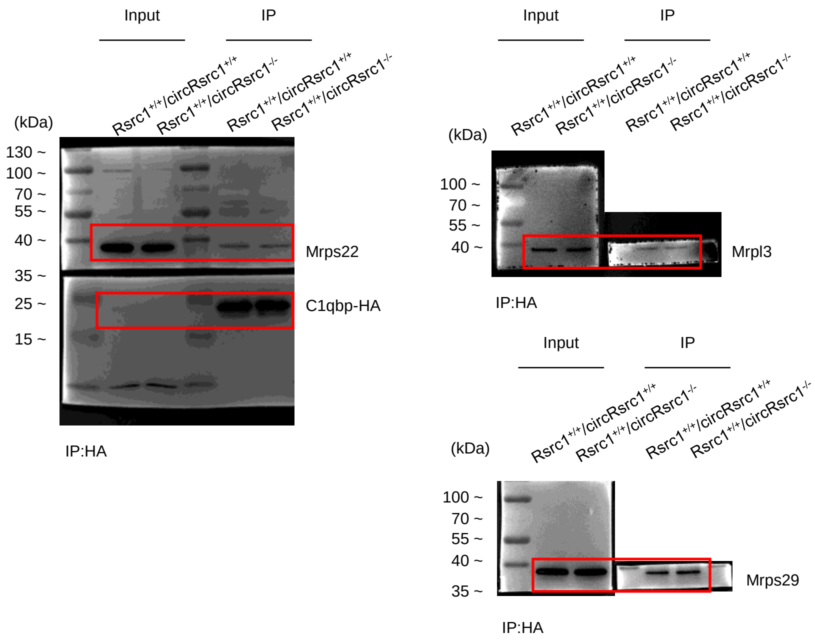

Fig. S7d

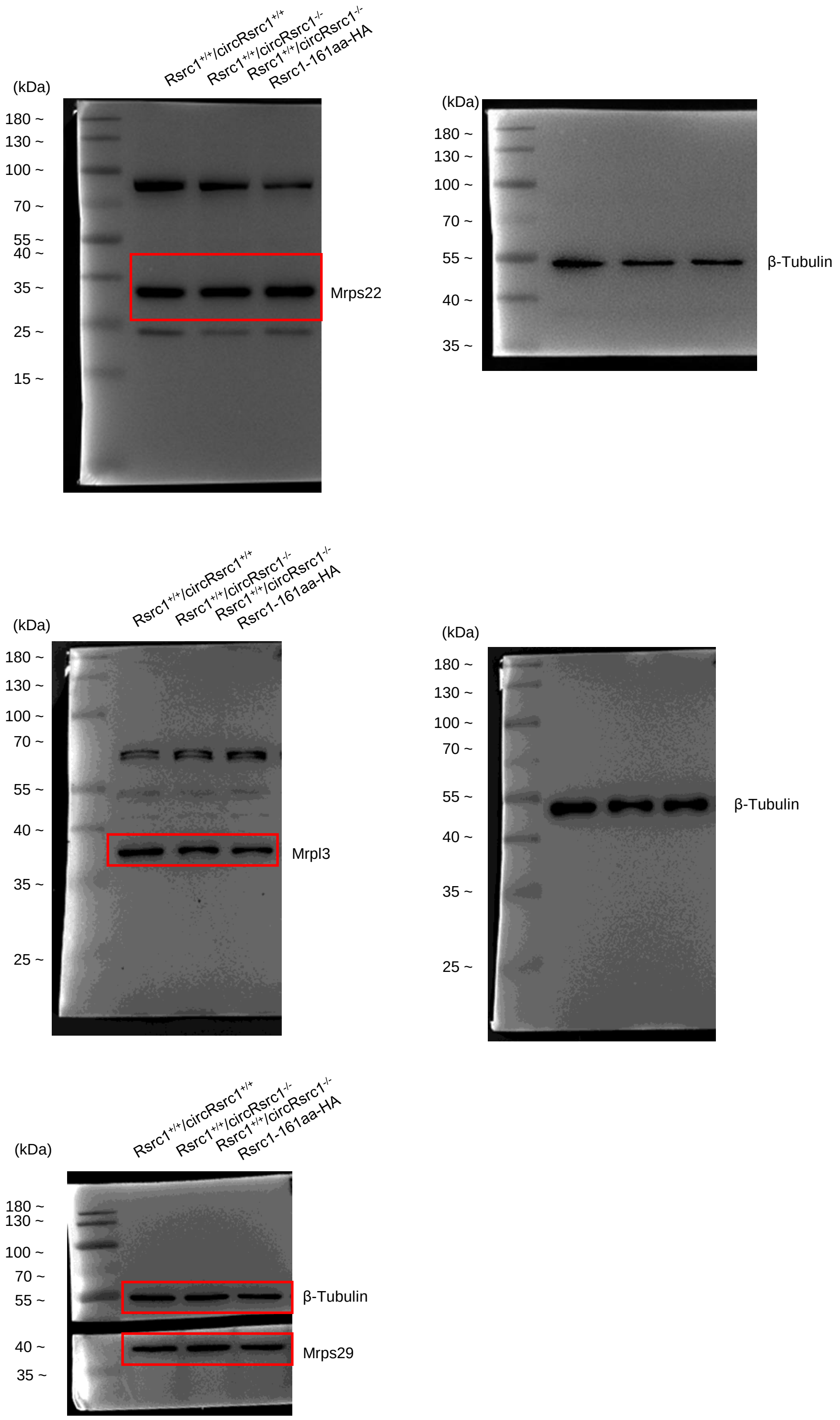
Rsrc1+/+/circRsrc1+/+
Rsrc1+/+/circRsrc1-/-
Rsrc1+/+/circRsrc1-/-
Rsrc1-161aa-HA
(kDa)
180 ~
130 ~
100 ~
70 ~
55 ~
40 ~
35 ~
25 ~
15 ~
Mrps22
(kDa)
180 ~
130 ~
100 ~
70 ~
55 ~
40 ~
35 ~
β-Tubulin
Rsrc1+/+/circRsrc1+/+
Rsrc1+/+/circRsrc1-/-
Rsrc1+/+/circRsrc1-/-
Rsrc1-161aa-HA
(kDa)
180 ~
130 ~
100 ~
70 ~
55 ~
40 ~
35 ~
25 ~
Mrpl3
(kDa)
180 ~
130 ~
100 ~
70 ~
55 ~
40 ~
35 ~
25 ~
β-Tubulin
Rsrc1+/+/circRsrc1+/+
Rsrc1+/+/circRsrc1-/-
Rsrc1+/+/circRsrc1-/-
Rsrc1-161aa-HA
(kDa)
180 ~
130 ~
100 ~
70 ~
55 ~
40 ~
35 ~
β-Tubulin
Mrps29
